# Testing Spatial Symmetry Using Contingency Tables Based on Nearest Neighbor Relations

**DOI:** 10.1155/2014/698296

**Published:** 2014-01-19

**Authors:** Elvan Ceyhan

**Affiliations:** Department of Mathematics, Koç University, Sarıyer, 34450 Istanbul, Turkey

## Abstract

We consider two types of spatial symmetry, namely, symmetry in the mixed or shared nearest neighbor (NN) structures. We use Pielou's and Dixon's symmetry tests which are defined using contingency tables based on the NN relationships between the data points. We generalize these tests to multiple classes and demonstrate that both the asymptotic and exact versions of Pielou's first type of symmetry test are extremely conservative in rejecting symmetry in the mixed NN structure and hence should be avoided or only the Monte Carlo randomized version should be used. Under RL, we derive the asymptotic distribution for Dixon's symmetry test and also observe that the usual independence test seems to be appropriate for Pielou's second type of test. Moreover, we apply variants of Fisher's exact test on the shared NN contingency table for Pielou's second test and determine the most appropriate version for our setting. We also consider pairwise and one-versus-rest type tests in post hoc analysis after a significant overall symmetry test. We investigate the asymptotic properties of the tests, prove their consistency under appropriate null hypotheses, and investigate finite sample performance of them by extensive Monte Carlo simulations. The methods are illustrated on a real-life ecological data set.

## 1. Introduction

The analysis of spatial point patterns in natural populations (in ℝ^2^ and ℝ^3^) has been studied extensively. In particular, spatial patterns in epidemiology, population biology, and ecology have important practical consequences. Since the early days of this research, most of the research has been on data from one class, that is, on spatial pattern of one class with respect to the ground (e.g., intensity, clustering, etc). An example of a pattern in a one-class framework is *aggregation* [1]. It is also of practical importance to investigate the spatial interaction between two or more classes, for example, spatial patterns of one class with respect to other classes [2]. Two frequently studied spatial patterns between multiple classes or species are segregation and association. Segregation occurs when an individual is more likely to be found near conspecifics (i.e., individuals of the same species) [3] and association occurs when an individual from one class is more likely to be found near individuals from the other class.

There are many tests available in the literature for the analysis of spatial point patterns in various fields. An extensive survey is provided by Kulldorff [4] who enumerates more than 100 such tests, most of which need adjustment for some sort of inhomogeneity. However, none of the tests surveyed by Kulldorff [4] are designed for testing spatial symmetry. Most of the tests for multiple classes deal with the existence (or lack) of spatial interaction (in the form of spatial association or segregation) between the classes.

In the literature, Baczkowski and Mardia [5] proposed methods for testing spatial symmetry based on the sample semivariogram. Their methods are applicable for a Gaussian doubly geometric process on a regular lattice. The latest methods for testing and detecting isotropy, symmetry, and separability in spatiotemporal models are discussed in a recent book by Sherman [6] who investigated these properties in the directional sense. For example, isotropy is assessed in the sense of direction-independence of the second-order properties of the spatial point pattern. Spatial symmetry is not only useful in ecological contexts (as in spatial symmetry of plant species in a region of interest), but also in socioeconomic theory to help understand spatial equilibrium configurations [7]. Axial symmetry methods based on the sample periodogram for data collected on a rectangular lattice are also considered and shown to perform well in Scaccia and Martin [8].

The methods discussed in the current paper are the spatial symmetry tests based on NN relationships. There are at least six different groups of NN methods for spatial patterns (see, e.g., [9]). These methods are based on some measure of (dis)similarity between a point and its NN, such as the distance between the point and its NN or the class types of the point and its NN. For example, Pielou [2] constructed nearest neighbor contingency tables (NNCTs) which yield tests of segregation (positive or negative), symmetry, and niche specificity, and a coefficient of segregation in a two-class setting. Additionally, Dixon devised overall, class- and cell-specific tests based on NNCTs for the two-class case in [10] and extended his methodology to multiclass case in [3]. Pielou's and Dixon's symmetry tests are designed to detect the symmetry (or lack of it) in the mixed or shared NN structure and are the only tests for detecting such symmetry structure (to the authors knowledge). Symmetry in mixed NN structure implies equality of the expected values of the number of NN pairs in which the points in the pair are from different classes, while symmetry in shared NN structure implies that the proportion—with respect to the class size—of number of times points from one class serving as NN to other classes is equal for all classes. Asymmetry in mixed NN structure would be suggestive of different types or levels of spatial interaction between the two classes of points, while asymmetry in shared NN structure would indicate differences in spatial distribution of points from one class with respect to all the points (from both classes) in the study region compared to that of points from the other class.

Pielou has described her symmetry tests for completely mapped data in ℝ^2^, although her tests are not appropriate for such data [10, 11]. A data set is *completely mapped*, if the locations of all events in a defined space are observed. We assume that data is *sparsely sampled*; that is, only a (random) subset of NN pairs is observed for Pielou's first type of symmetry test. Pielou's first type of symmetry and Dixon's symmetry test are based on the NNCTs that are constructed using the NN frequencies. Both tests are defined for the two-class case only. Pielou's second type of symmetry test is based on the frequencies of number of times points in a class serve as NNs yielding a contingency table which we call *Q-symmetry contingency table*. So, points from each class are categorized into six groups, namely 0,1,…, 5, where a point serving as a NN to no other point is in category “0,” to one other point is in category “1,” and so on. Due to geometric constraints, in ℝ^2^, a point can not serve as a NN to more than six points. For data from a continuous distribution, a point can serve as a NN to at most five points almost surely. Under spatial symmetry in shared NN structure in a multiclass case, the frequencies of these six categories should have the same distributional form for each class.

Pielou's symmetry tests were introduced and illustrated in [2], while Dixon's symmetry test was introduced in passing in [10]. None of tests were extensively studied nor investigated for size and/or power performance. In this paper, we investigate the underlying assumptions for these symmetry tests. We derive their asymptotic distributions under appropriate null hypotheses and extend these symmetry tests to multiclass case. In particular, we demonstrate that Pielou's first type of symmetry test is extremely conservative when used as McNemar's test with its asymptotic critical value and hence should be avoided in practice (or its Monte Carlo randomized version can be used). We also show that various patterns can constitute as the null case for Dixon's symmetry test and Pielou's second type of symmetry test but derive the asymptotic distributions of these tests under CSR independence and RL patterns only. We also investigate the use of Fisher's exact test on the *Q*-symmetry contingency table used for Pielou's second type of symmetry test (for shared NN structure). Moreover, the tests discussed in this paper are constructed using the NN relations based on the usual Euclidean distance; so we discuss the generalization of the tests for the case in which NN relations are defined by a dissimilarity measure. Furthermore, we discuss the extension of the methodology to high or infinite dimensional data. In a multiclass setting, first the overall symmetry is tested and if the overall test is significant we propose various post hoc tests such as pairwise symmetry tests or one-versus-rest type tests. The local asymptotic power of the tests is also investigated using the local approximation of the power function or Pitman asymptotic efficiency. Finite sample empirical (size and power) performance comparisons are investigated by Monte Carlo simulations.

We describe and discuss switch the order of these two parts, tests of symmetry in NN structure, their extension to multiclass case, and the corresponding sampling frameworks for the cell counts (i.e., entries in the contingency tables) in Section 2. We discuss the variants of Fisher's exact test for the *Q*-symmetry contingency table in Section 3 and asymptotic power analysis (i.e., consistency of the tests and their asymptotic efficiency) in Section 4 and provide an extensive empirical performance analysis by Monte Carlo simulations in Section 5. We discuss the use of one-versus-rest and pairwise tests as post hoc tests in Section 6, illustrate the methodology on an ecological data set in Section 7, discuss the extension of the methodology to the case where NN relations are defined with dissimilarity measures in Section 8, and provide some guidelines and discussion in Section 9.

## 2. Tests of Symmetry in the NN Structure

Two or more classes may exhibit many different forms of spatial asymmetry. Although it is not possible to list all possible asymmetry types or configurations, existence of asymmetry can be detected by an analysis of the NN relationships of the class members.

### 2.1. Preliminaries

The null case for asymmetry alternatives is that there is symmetry in the allocations of points with respect to each other. In particular, consider symmetry in mixed NN structure for two classes *i* and *j*. Then the null case is that the expected number of times class *i* points serving as NN to class *j* would be the same as the expected number of times class *j* points serving as NN to class *i*. On the other hand, for symmetry in shared NN structure, the vector of relative frequencies (with respect to the class size) of points from each class serving as NN to other points is the same for all classes. In general, the null hypothesis for symmetry in mixed NN structure would be implied by a more general pattern, namely, if there is randomness in the NN structure in such a way that the probability of a NN of a point being from a class is proportional to the relative frequency of that class. This assumption holds, for example, under RL or CSR independence of the points from each class. Under CSR independence, the points from each class are independent realizations of homogeneous Poisson process (HPP) with fixed class sizes. In particular, conditioned on the class sizes, the points are independently uniformly distributed in the region of interest. Under RL, class labels are independently and randomly assigned to a set of given locations, where these locations could be from HPP or some other clustered or regular pattern. The null hypothesis for symmetry in shared NN structure would be implied if there is randomness in the NN structure in such a way that the probability of a point from a class serving as NN to *m* other points is proportional to the relative frequency of that class. This assumption also holds under RL or CSR independence of the points from each class. Therefore, both CSR independence and RL patterns would imply symmetry in the mixed or shared NN structure.

Pielou suggests two types and Dixon suggests one type of symmetry tests in the two-class case. Pielou's first type of symmetry test and Dixon's symmetry test are defined for the two-class case only and are based on the corresponding 2 × 2 NNCT. We provide a brief description of NNCTs; for a more detailed discussion see, for example, [12]. Suppose that there are *k* = 2 classes labeled as {1,2}. NNCTs are constructed using NN frequencies for each class. Let *n*_*i*_ be the number of points from class *i* for *i* ∈ {1,2} and *n* = *n*_1_ + *n*_2_. If we record the class of each point and its NN, the NN relationships fall into *k* ^2^ = 4 categories: (1,1), (1,2); (2,1), (2,2), where in category (*i*, *j*), class *i* is the base class and class *j* is the class of the NN. Denoting *N*_*ij*_ as the observed frequency of category (*i*, *j*) for *i*, *j* ∈ {1,2}, we obtain the NNCT in Table 1, where *C*_*j*_ is the sum of column *j*; that is, a number of times class *j* points serve as NNs for *j* ∈ {1,2}. Note also that *n*_*i*_ = ∑_*j*=1_^2^ *N*_*ij*_, *C*_*j*_ = ∑_*i*=1_^2^ *N*_*ij*_, and *n* = ∑_*i*,*j*_ *N*_*ij*_ = ∑_*i*=1_^2^ *n*_*i*_ = ∑_*j*=1_^2^ *C*_*j*_. Throughout the paper, we adopt the convention that random variables are denoted with upper case letters and fixed quantities with lower case letters. Notice that row sums (i.e., class sizes) are assumed to be fixed, while column sums (i.e., number of times a class serves as NN) is random in our NNCTs.

### 2.2. Pielou's First Type of Symmetry Test

Pielou's first type of symmetry test involves testing equality of expected values of mixed NN frequencies, that is, the equality of expected values of off-diagonal entries in the NNCT. So Pielou's first type of symmetry test is used to detect the symmetry in the “mixed NN structure.” In this case, if *N*_12_ ≈ *N*_21_, spatial allocation of points from two classes is *symmetric* with respect to the (mixed) NN structure; otherwise, the population is *asymmetric*. When two classes, *X* and *Y*, are of equal size, in a symmetric population, points from each class are equally likely to serve as NN to points from the other class, and, in an asymmetric population, points from one class, say class *X*, tend to serve more as NN to points from class *Y* compared to class *Y* serving as NN to points from class *X*. So the null hypothesis is

(1) $$\begin{array}{c} H_{o}:E\left[N_{12}\right]=E\left[N_{21}\right] \end{array}$$

which may have various forms based on the assumed underlying frameworks for the contingency tables in general and for the NNCTs.

The two-sided alternative is usually a more reasonable alternative, although one-sided alternatives are also possible. Pielou [2] tests for significant differences between *N*_12_ and *N*_21_ with a *χ* ^2^ test (with Yates' correction) with 1 df using

(2) $$\begin{array}{c} {𝒳}_{\text{I}}^{2}=\frac{{\left(\left|N_{12}-N_{21}\right|-1\right)}^{2}}{N_{12}+N_{21}} \end{array}$$

which is the same as the McNemar's test with continuity correction [13]. This test is appropriate only for sparsely sampled data and large *N*_12_ + *N*_21_ with neither *N*_12_ or *N*_21_ being too small compared to each other and applicable only for the two-class case. So we suggest the approach recommended in Remark 1 below. The discussion till the end of this subsection is for (properly) sparsely sampled data. Furthermore, in a population in which two classes are highly segregated or the intensities (number of points per unit area) of the classes are very different, the frequencies *N*_12_ and *N*_21_ can be too small, which renders the *χ* ^2^ approximation inappropriate for the test in (2). In such a case, one can use the exact finite sample distribution of *N*_12_ which follows a binomial distribution (conditionally). Given that *N*_12_ + *N*_21_ = *n*_*t*_, the test statistic *N*_12_ has a BIN(*n*_*t*_, 1/2) distribution under *H*_*o*_ for properly sparsely sampled data, where BIN(*n*, *p*) stands for the binomial distribution with *n* independent trials with probability of success *p*. So, for small *n*_*t*_, the statistic, *N*_12_, can be used with the binomial critical values. For large *n*_*t*_,

(3) $$\begin{array}{c} Z_{\text{I}}=\frac{N_{12}-n_{t}/2}{\sqrt{n_{t}/4}}=\frac{N_{12}-N_{21}}{\sqrt{N_{12}+N_{21}}} \end{array}$$

has approximately *N*(0,1) distribution, so *Z*_I_ can be used for the one-sided alternatives. Furthermore, the test *𝒳*_I_^2^ in (2) has approximately *χ*_1_^2^ distribution, which can only be used for the two-sided alternative.

Pielou's first type of symmetry test can be extended to the multiclass case (with *k* > 2) as

(4) $$\begin{array}{c} {𝒳}_{\text{I}}^{2}=\underset{i<j}{\sum }\frac{{\left(\left|N_{ij}-N_{ji}\right|-1\right)}^{2}}{N_{ij}+N_{ji}}. \end{array}$$

Under *H*_*o*_: “*p*_*ij*_ = *p*_*ji*_, for all *i* ≠ *j*”, *𝒳*_I_^2^ in (4) is the same as Bowker's test of symmetry, which is an extension of McNemar's test for the *k* × *k* contingency tables [14]. The test statistic in this case has *χ*_*k*(*k*−1)/2_^2^ distribution asymptotically.

#### 2.2.1. The Row-Wise Multinomial Framework

In general, a contingency table may result from various frameworks. The first type of framework is the *row-wise multinomial framework*, where each row in a *k* × *k* contingency table is independent of other rows and is from a multinomial distribution. That is, letting the entries of the contingency table be denoted as *N*_*ij*_ (as in the NNCT in Table 1), we have entries in row *i* having (*N*_*i*1_, *N*_*i*2_,…, *N*_*ik*_) ~ *ℳ*(*n*_*i*_, *p*_*i*1_, *p*_*i*2_,…, *p*_*ik*_), where *p*_*ij*_ is the probability of an experimental unit being from row category *i* and column category *j* simultaneously and *ℳ*(*n*, *p*_1_, *p*_2_,…, *p*_*k*_) standing for the multinomial distribution with *n* independent trials and the probability of a trial resulting in category *k* is *p*_*k*_ with ∑_*i*=1_^*k*^ *p*_*i*_ = 1. In the 2 × 2 contingency table, the rows will have two entries, so the multinomial distribution reduces to a binomial distribution. More specifically, we would have *N*_*i*1_ ~ BIN(*n*_*i*_, *p*_*i*1_) (or *N*_*i*2_ ~ BIN(*n*_*i*_, *p*_*i*2_)) for *i* = 1,2.

In a NNCT, *k* is the number of classes and *p*_*ij*_ is the probability of a point from class *j* serving as a NN to a point from class *i* for *i*, *j* ∈ {1,2,…, *k*}. However, a NNCT is unlikely to result from a row-wise multinomial framework. In a NNCT, a trial is the categorization of a base-NN pair; that is, a trial is “determining the type of a base-NN pair.” For entry (*i*, *j*), a trial results in success, if a base-NN pair belongs to category *i*, *j* (i.e., base point is from class *i* and its NN point is from class *j*). For example, in a 2 × 2 contingency table, in general, (*N*_11_, *N*_12_) and (*N*_21_, *N*_22_) are assumed to be independent and so are the individual trials under the row-wise multinomial framework. This assumption is invalid when the NNCT is based on completely mapped data because independence between rows is violated (see also Remark 1). If the NNCT is constructed using a random sample of base-NN pairs, then the usual contingency table assumptions under the row-wise multinomial framework would hold. Such a NNCT can be (approximately) obtained only if a (small) subset of all the base-NN pairs obtained from the data in the study region was randomly selected; that is, if the data is obtained by an appropriate sparse sampling. When the data were properly sparsely sampled, we will assume that the NNCT satisfies the usual independence assumptions in the row-wise multinomial framework henceforth. In this framework, the explicit form of the null hypothesis becomes

(5) $$\begin{array}{c} H_{o}:n_{1}p_{12}=n_{2}p_{21}. \end{array}$$

When the 2 × 2 NNCT is constructed from a sparsely sampled data, the rows are assumed to be from the same multinomial distribution, so the entries in row *i* satisfy *N*_*ij*_ ~ BIN(*n*_*i*_, *κ*_*j*_) for *j* = 1,2, where *κ*_*j*_ is the probability of a NN point being from class *j*. Then, under *H*_*o*_ in (5), we have **E**[*N*_12_] = **E**[*N*_21_], which holds if and only if *n*_1_ *p*_12_ = *n*_2_ *p*_21_ if and only if *n*_1_ *κ*_2_ = *n*_2_ *κ*_1_. Since *κ*_1_ + *κ*_2_ = 1 in a two-class setting, we have *n*_1_(1 − *κ*_1_) = *n*_2_ *κ*_1_ if and only if *n*_1_ = (*n*_1_ + *n*_2_)  *κ*_1_ = *nκ*_1_. Letting *ν*_*i*_ be the proportion of points from class *i* in our sample, we have *nν*_1_ = *nκ*_1_ if and only if *ν*_1_ = *κ*_1_. One-sided or two-sided alternatives are possible for the *H*_*o*_ in (5).

#### 2.2.2. The Overall Multinomial Framework

An alternative framework for a general contingency table is the *overall multinomial framework*. In this case, the cell counts are assumed to arise from independent multinomial trials. That is, for example, for a *k* × *k* contingency table,

(6) N=(N11,N12,…,N1k,N21,N22,…,N2k,Nk1, Nk2,…,Nkk)~ℳ(n,p11,p12,…,p1k,p21,p22,…,p2k,…,   pk1,pk2,…,pkk).

For a NNCT, if the data is completely mapped, independence between trials is violated again. Under sparse sampling, this framework is able to model a NNCT approximately. That is, if the NNCT is based on a random sample of base-NN pairs, it will (approximately) satisfy the assumptions in the overall multinomial framework because of the inherent correlation between components or entries of a multinomially distributed random variable. For example, in a two-class setting with sparsely sampled data, we have

(7) $$\begin{array}{c} N=\left(N_{11},N_{12},N_{21},N_{22}\right)\sim ℳ\left(n,\nu_{1}\kappa_{1},\nu_{1}\kappa_{2},\nu_{2}\kappa_{1},\nu_{1}\kappa_{2}\right), \end{array}$$

where *ν*_1_ + *ν*_2_ = 1 and *κ*_1_ + *κ*_2_ = 1. Then the null hypothesis of symmetry becomes

(8) $$\begin{array}{c} H_{o}:p_{12}=p_{21} \end{array}$$

which is equivalent to *H*_*o*_ : *ν*_1_ *κ*_2_ = *ν*_2_ *κ*_1_ or equivalently *H*_*o*_ : *ν*_1_ = *κ*_1_ since *κ*_2_ = 1 − *κ*_1_ and *ν*_2_ = 1 − *ν*_1_. Row-wise and overall multinomial frameworks are closely related. Conditional on *N*_*i*_ = *n*_*i*_, the overall multinomial framework reduces to the row-wise multinomial framework. But a NNCT for completely mapped data does not fit to the overall multinomial framework either, due to the inherent spatial dependence and the row sums being fixed. *McNemar's test (and hence Bowker's test) is only appropriate in the overall multinomial framework and is extremely conservative for the row-wise multinomial framework.* In particular, in a NNCT for completely mapped data, we have row sums (i.e., class sizes) fixed as in the row-wise multinomial framework. Hence, *Pielou's first type of symmetry test would also be extremely conservative for the NNCTs*.

**Remark**

In Pielou's first type of symmetry test, both of the above multinomial frameworks assume that the trials are independent multinomial trials. However, when a trial is the base-NN relation, the assumption of independence between trials is violated. The dependence mainly originates from the fact that a point is more likely to be the NN of its own NN (i.e., more likely to form a *reflexive* (base, NN) pair); hence, many reflexive pairs are possible. Thus, Pielou's test is influenced by deviations not only from the null case but also by deviations from dependence on trials. The dependence due to reflexivity can not merely be avoided by random subsampling but can be circumvent by an appropriate sparse sampling [15]. The assessment of various sparse sampling schemes for these tests is a topic of ongoing research. Furthermore, Pielou's first type of symmetry test requires the NNCT resulting from an overall multinomial framework, which does not hold for a NNCT based on completely mapped data either. So Pielou's first type of symmetry test is only appropriate under the overall multinomial framework (with random row sums), which can be satisfied by an appropriate sparse sampling. *Our suggestion for Pielou's first type of symmetry test is as follows. If the data is properly sparsely sampled under the overall multinomial framework, then one can employ it. But if the data is completely mapped, to remove the influence of spatial dependence on Pielou's first type of symmetry test, we suggest the usual Monte Carlo randomization*, where class labels are randomly assigned to the given points a large number of times and test statistics are computed, and the *p* value of the test is based on the rank (scaled by the number of Monte Carlo replications) of the test statistic of the original data in the sample of test statistics obtained from Monte Carlo randomization procedure.

### 2.3. Dixon's Symmetry Test

Dixon [10] also suggested a symmetry test for testing the equality of frequency of mixed NNs (or between class NNs), that is, the equality of the expected values of the off-diagonal entries in the 2 × 2 NNCTs. So the null hypothesis is given by

(9) $$\begin{array}{c} H_{o}:E\left[N_{12}\right]=E\left[N_{21}\right] \end{array}$$

and, under RL or CSR independence, **E**[*N*_*ij*_] = *n*_*i*_ *n*_*j*_/*n* for *i* ≠ *j*. Notice that the null hypotheses for Dixon's symmetry test and Pielou's first type of symmetry test look identical; however, the corresponding underlying assumptions are different. Pielou's first type of symmetry test is only appropriate when the data are (properly) sparsely sampled under overall multinomial framework, whereas Dixon's symmetry test is appropriate when the data are completely mapped. That is, Pielou's first type of symmetry test is appropriate when we have a random sample of base-NN pairs under overall multinomial framework; that is, between-row independence assumptions are satisfied (up to the inherent correlation for multinomial entries) and, hence, ignore the spatial information. On the other hand, Dixon's test can be used for completely mapped data and takes the spatial dependence into account.

Under RL, the test statistic for Dixon's symmetry test is given by

(10) ZD:=N12−N21−E[N12−N21]Var[N12−N21]=N12−N21Var[N12]+Var[N21]−2Cov[N12,N21],

where **E**[*N*_12_ − *N*_21_] = **E**[*N*_12_] − **E**[*N*_21_] = 0 since **E**[*N*_12_] = **E**[*N*_21_] = *n*_1_ *n*_2_/2 and **V** **a** **r**[*N*_12_ − *N*_21_] = **V** **a** **r**[*N*_12_] + **V** **a** **r**[*N*_21_] − 2**C** **o** **v**[*N*_12_, *N*_21_] with

(11) Var[Nij]=npij+Qpiij+(n2−3n−Q+R)piijj−(npij)2

for (*i*, *j*)∈{(1,2), (2,1)} and

(12) Cov[N12,N21]=Rp12+(n−R)(p112+p122)+(n2−3n−Q+R)p1122−n2p12p21.

Here *p*_*xx*_, *p*_*xxx*_, and *p*_*xxxx*_ are the probabilities that a randomly picked pair, triplet, or quartet of points, respectively, are from the indicated classes and are given by

(13) $$\begin{array}{c} p_{ii}=\frac{n_{i}\left(n_{i}-1\right)}{n\left(n-1\right)},\\ p_{iii}=\frac{n_{i}\left(n_{i}-1\right)\left(n_{i}-2\right)}{n\left(n-1\right)\left(n-2\right)},\\ p_{iiii}=\frac{n_{i}\left(n_{i}-1\right)\left(n_{i}-2\right)\left(n_{i}-3\right)}{n\left(n-1\right)\left(n-2\right)\left(n-3\right)},\\ p_{ij}=\frac{n_{i}n_{j}}{n\left(n-1\right)},\\ p_{iijj}=\frac{n_{i}\left(n_{i}-1\right)n_{j}\left(n_{j}-1\right)}{n\left(n-1\right)\left(n-2\right)\left(n-3\right)}. \end{array}$$

Furthermore, *Q* is the number of points with shared NNs, which occurs when two or more points share a NN and *R* is twice the number of reflexive pairs. Then *Q* = 2(*Q*_2_ + 3*Q*_3_ + 6*Q*_4_ + 10*Q*_5_ + 15*Q*_6_), where *Q*_*j*_ is the number of points that serves as a NN to other points *j* times. For large *n*_*i*_, *Z*_*D*_ asymptotically has *N*(0,1) distribution. A two-sided alternative and one-sided alternatives are possible with the test statistic, *Z*_*D*_.

We describe this setting in a broader context with *k* ≥ 2 classes. Let *ν*_*i*_ be the probability of an arbitrary point being from class *i* and *ν*_*ij*_ be the probability of a base-NN pair with base point being from class *i* and its NN being from class *j*. Then, under RL, we have *ν*_*ij*_ = *ν*_*i*_ *ν*_*j*_ and the expression (*n*_*i*_(*n*_*i*_ − 1)/*n*(*n* − 1))**I**  (*i* = *j*)+(*n*_*i*_ *n*_*j*_/*n*(*n* − 1))**I**  (*i* ≠ *j*) can be viewed as an estimator or approximation for *ν*_*ij*_ for large *n*_*i*_, where **I**(·) stands for the indicator function. Furthermore, for large *n*_*i*_, the null hypothesis of symmetry is equivalent to

(14) $$\begin{array}{c} H_{o}:E\left[N_{12}\right]=E\left[N_{21}\right]\approx n\nu_{1}\nu_{2}. \end{array}$$

In Dixon's framework, for large *n*_*i*_, the row marginals satisfy *n*_*i*_/*n* ≈ *ν*_*i*_ and the column marginals satisfy *κ*_*j*_ = **E**[*C*_*j*_/*n*] = ∑_*i*=1_^*k*^ *ν*_*i*_ *ν*_*j*_ = *ν*_*j*_.

The symmetry in mixed NN frequencies may result from various patterns. In particular, under RL or CSR independence, *H*_*o*_ in (9) would hold. In a RL framework with fixed allocation of points, the quantities *Q* and *R* are also fixed, but, for a CSR allocation of points, *Q* and *R* are random with **E**[*Q*/*n*]≈.63 and **E**[*R*/*n*]≈.62 for large *n* (estimated empirically by Monte Carlo simulations for homogeneous planar Poisson pattern). Hence, *Z*_*D*_ in (10) and its distribution is conditional on *Q* and *R* under CSR independence but unconditional under RL. To be more precise, under CSR independence, the expected values for *Z*_*D*_ are as in RL, but the variances and covariances are conditional on *Q* and *R*. Given the difficulty in finding the distribution of *Q* and *R* under CSR, we use their observed values even if the null hypothesis is implied by CSR independence.

#### 2.3.1. Extension of Dixon's Symmetry Test to Multiclass Case

Dixon's symmetry test can be extended to the *k* > 2 case as follows. Consider the *N*_*ij*_ − *N*_*ji*_ values for *i* < *j*. Combining *N*_*ij*_ − *N*_*ji*_ values for *i* < *j*, we obtain the vector

(15) TS(N12−N21,N13−N31,…,N1k−Nk1, N23−N32,…,N2k−Nk2,…,N(k−1)k−Nk(k−1))

which has length *k*(*k* − 1)/2. Under RL, **E**[*N*_*ij*_ − *N*_*ji*_] = **E**[*N*_*ij*_] − **E**[*N*_*ji*_] = *n*_*i*_ *n*_*j*_/*n* − *n*_*j*_ *n*_*i*_/*n* = 0 and **V** **a** **r**[*N*_*ij*_ − *N*_*ji*_] = **V** **a** **r**[*N*_*ij*_] + **V** **a** **r**[*N*_*ji*_] − 2**C** **o** **v**[*N*_*ij*_, *N*_*ji*_], where **C** **o** **v**[*N*_*ij*_, *N*_*ji*_] = *Rp*_*ij*_ + (*n* − *R*)(*p*_*iij*_ + *p*_*ijj*_)+(*n* ^2^ − 3*n* − *Q* + *R*)*p*_*ii*_ *p*_*jj*_ − *n* ^2^ *p*_*ij*_ *p*_*ji*_. For large *n*_*i*_,

(16) $$\begin{array}{c} Z_{D}^{ij}=\frac{N_{ij}-N_{ji}}{\sqrt{Var\left[N_{ij}-N_{ji}\right]}} \end{array}$$

approximately has *N*(0,1) distribution.

To combine the entries of the vector **T**_*S*_ in one overall test statistic for symmetry, we also need the covariance matrix of **T**_*S*_, denoted Σ_sym_. The diagonal entries of **T**_*S*_ are **V** **a** **r**[*N*_*ij*_ − *N*_*ji*_] in the order of entries of **T**_*S*_. For the off-diagonal entries, we need the covariance terms **C** **o** **v**[*N*_*ij*_ − *N*_*ji*_, *N*_*kl*_ − *N*_*lk*_]. By construction, we have *i* < *j* and *k* < *l* and there are six cases regarding these covariance terms.

*Case  1* (*i* = *k* and *j* = *l*). In this case, the covariance term is just the variance term, **V** **a** **r**[*N*_*ij*_ − *N*_*ji*_].

*Case  2* (*i* = *k* and *j* ≠ *l*). **C** **o** **v**[*N*_*ij*_ − *N*_*ji*_, *N*_*il*_ − *N*_*li*_] = **C** **o** **v**[*N*_*ij*_, *N*_*il*_] − **C** **o** **v**[*N*_*ij*_, *N*_*li*_] − **C** **o** **v**[*N*_*ji*_, *N*_*il*_] + **C** **o** **v**[*N*_*ji*_, *N*_*li*_]. 

*Case  3* (*i* ≠ *k* and *j* = *l*). **C** **o** **v**[*N*_*ij*_ − *N*_*ji*_, *N*_*kj*_ − *N*_*jk*_] = **C** **o** **v**[*N*_*ij*_, *N*_*kj*_] − **C** **o** **v**[*N*_*ij*_, *N*_*jk*_] − **C** **o** **v**[*N*_*ji*_, *N*_*kj*_] + **C** **o** **v**[*N*_*ji*_, *N*_*jk*_].

*Case  4* (*i* = *l* and *j* ≠ *k*). **C** **o** **v**[*N*_*ij*_ − *N*_*ji*_, *N*_*ki*_ − *N*_*ik*_] = **C** **o** **v**[*N*_*ij*_, *N*_*ki*_] − **C** **o** **v**[*N*_*ij*_, *N*_*ik*_] − **C** **o** **v**[*N*_*ji*_, *N*_*ki*_] + **C** **o** **v**[*N*_*ji*_, *N*_*ik*_].

*Case  5* (*i* ≠ *l* and *j* = *k*). **C** **o** **v**[*N*_*ij*_ − *N*_*ji*_, *N*_*jl*_ − *N*_*lj*_] = **C** **o** **v**[*N*_*ij*_, *N*_*jl*_] − **C** **o** **v**[*N*_*ij*_, *N*_*lj*_] − **C** **o** **v**[*N*_*ji*_, *N*_*jl*_] + **C** **o** **v**[*N*_*ji*_, *N*_*lj*_].

*Case  6* (*i* ≠ *k*, *i* ≠ *l* and *j* ≠ *l*, *j* ≠ *k*). **C** **o** **v**[*N*_*ij*_ − *N*_*ji*_, *N*_*kl*_ − *N*_*lk*_] = **C** **o** **v**[*N*_*ij*_, *N*_*kl*_] − **C** **o** **v**[*N*_*ij*_, *N*_*lk*_] − **C** **o** **v**[*N*_*ji*_, *N*_*kl*_] + **C** **o** **v**[*N*_*ji*_, *N*_*lk*_].

The covariance term in Case 6 above is zero, since

(17) $$\begin{array}{c} Cov\left[N_{ij},N_{kl}\right]=\left(n^{2}-3n-Q+R\right)p_{ijkl}-n^{2}p_{ij}p_{kl},\\ Cov\left[N_{ij},N_{lk}\right]=\left(n^{2}-3n-Q+R\right)p_{ijlk}-n^{2}p_{ij}p_{lk},\\ Cov\left[N_{ji},N_{kl}\right]=\left(n^{2}-3n-Q+R\right)p_{jikl}-n^{2}p_{ji}p_{kl},\\ Cov\left[N_{ji},N_{lk}\right]=\left(n^{2}-3n-Q+R\right)p_{jilk}-n^{2}p_{ji}p_{lk},\\ p_{ijkl}=p_{ijlk}=p_{jikl}=p_{jilk}=\frac{n_{i}n_{j}n_{k}n_{l}}{n\left(n-1\right)\left(n-2\right)\left(n-3\right)},\\ p_{ij}p_{kl}=p_{ij}p_{lk}=p_{ji}p_{kl}=p_{ji}p_{lk}=\frac{n_{i}n_{j}}{n\left(n-1\right)}\frac{n_{k}n_{l}}{n\left(n-1\right)}. \end{array}$$

Notice that Σ_sym_ is a *k*_*s*_ × *k*_*s*_ matrix with *k*_*s*_ = *k*(*k* − 1)/2 and **E**[**T**_*S*_] = (0,0,…, 0). Then

(18) $$\begin{array}{c} {𝒳}_{D}^{2}={\left(T_{S}-E\left[T_{S}\right]\right)}^{\prime }\Sigma_{\text{sym}}^{-}\left(T_{S}-E\;\left[T_{S}\right]\right)=T_{S}^{\prime }\Sigma_{\text{sym}}^{-}T_{S} \end{array}$$

asymptotically has *χ*_*k*_*s*__^2^ distribution.

### 2.4. Pielou's Second Type of Symmetry Test

In the two-class case, a more elaborate test of symmetry due to Pielou [2] is based on a 2 × 6 contingency table, called the *Q-symmetry contingency table*, where the class of each observation and the number of times it serves as a NN are recorded. A point can only serve as a NN to 0,1, 2,3, 4, or 5 other observations due to geometric constraints in ℝ^2^ provided that the points are from a continuous distribution (as in CSR independence). For a two-class population, the observations are sorted into two sets of frequencies, namely, *Q*_*i*,0_, *Q*_*i*,1_,…, *Q*_*i*,5_, for *i* = 1,2, where *Q*_*i*,*m*_ is the frequency of class *i* observations serving as a NN to *m* other points for *m* ∈ {0,1, 2,3, 4,5}. So Pielou's second type of symmetry test uses more spatial information than just the categorization of base-NN relations. Notice that *Q*_*i*,*m*_ is also the number of class *i* points shared as a NN by *m* other points.

The corresponding contingency table for the two-class case is given in Table 2(a), where *Q*_*m*_ is the column sum, that is, the total number of points serving as a NN *m* times or the number of points shared as a NN by *m* other points for *m* ∈ {0,1, 2,3, 4,5}. Only if the allocation of the points from both populations is symmetric in terms of frequency of “serving as a NN” property, the expected proportions of classes 1 and 2 points serving as NNs *m* times will be the same for each *m* value. Hence, this type of symmetry refers to “symmetry in shared NN structure.” Let ${\vec{p}}_{i}=(p_{i,0},\ldots ,p_{i,5})$ be the vector of probabilities (or proportions) associated with row *i* for *i* = 1,2 in the *Q*-symmetry contingency table under the row-wise multinomial framework. In a *Q*-symmetry contingency table, sum of row *i* equals *n*_*i*_ (i.e., size of class *i*). Hence, *Q*-symmetry contingency table may not result from the overall multinomial framework, since row sums in a *Q*-symmetry contingency table are fixed for completely mapped data. Furthermore, under RL, column sums *Q*_*m*_ are fixed and hence can be denoted as *Q*_*m*_ = *q*_*m*_, but, under CSR independence, *Q*_*m*_ are random quantities.

Thus, the null hypothesis of symmetry in the shared NN structure is given by

(19)  Ho:p→1=p→2=(p0,p1,…,p5) or (p1,0,…,p1,5)=(p2,0,…,p2,5)=(p0,p1,…,p5).

In general, if the independence assumptions in the row-wise multinomial framework hold, we would have **E**[*Q*_*i*,*m*_] = *n*_*j*_ *Q*_*m*_/*n*. Then we may test the equality of proportions by using the usual Pearson's *χ* ^2^ test

(20) $$\begin{array}{c} {𝒳}_{\text{II}}^{2}=\overset{5}{\underset{m=0}{\sum }}\;\overset{2}{\underset{j=1}{\sum }}\frac{{\left(Q_{i,m}-E\left[Q_{i,m}\right]\right)}^{2}}{E\left[Q_{i,m}\right]} \end{array}$$

which has approximately a *χ*_5_^2^ distribution for large *n*. Under RL, although it would be possible for a point to serve as NN to 6 other points with a positive probability (depending on the fixed allocation of the points), we will only consider up to 5 (and combine 5 and 6 categories and treat them as one category). If these categories have nonnegligible counts, then the above discussion can easily be extended to the case that shared NN frequencies have 7 levels, and the corresponding test has *χ*_6_^2^ distribution for large *n*.

A conservative requirement for the cell frequencies in the contingency table is that no expected cell count is less than 1 and no more than 20% of the cell counts are less than 5 [16]. Otherwise, it is recommended to merge some of the categories. For the *Q*-symmetry contingency table, in practice, such a merging would usually be necessary for *m* ≥ 2, whence the dimension of the contingency table becomes 2 × 3 and df becomes 2. Large values of *𝒳*_II_^2^ indicate deviations from the null case. Hence, if the *p* value is significant, then the population can be assumed to be asymmetric in the shared NN structure in the sense that the distribution of the rows in the *Q*-symmetry contingency table would be different for the two classes; that is, there is significant asymmetry in the shared NN structure.

Pielou's second type of symmetry test can immediately be extended to the multiclass case. With *k* > 2, we record the frequency of class *i* members serving as NN *m* times in a *k* × 6 contingency table (merging cells when necessary which might be needed for *m* ≥ 2). Then we obtain the contingency table given in Table 2(b). In the *k*-class case, the null hypothesis is

(21) Ho:p→i=(pi,0,…,pi,5)=(p0,p1,…,p5)∀i∈{1,2,…,k}.

The corresponding test statistic *𝒳*_II_^2^ = ∑_*m*=0_^5^∑_*i*=1_^*k*^((*Q*_*i*,*m*_ − **E**[*Q*_*i*,*m*_])^2^/**E**[*Q*_*i*,*m*_]) would be approximately distributed as *χ*_5(*k*−1)_^2^ (and when columns are merged for *m* ≥ 2, we obtain a *k* × 3  *Q*-symmetry contingency table and the asymptotic distribution is *χ*_2(*k*−1)_^2^) for large *Q*_*m*_ and *n*_*i*_ provided that the independence assumptions in the row-wise multinomial framework hold.

This test seems to arise from the row-wise multinomial framework by construction, with the test statistic, *𝒳*_II_^2^, given in (20). Furthermore, the trials here are “base point-” “number of times the point serving as a NN” or “base point-” “number of times the point is shared as a NN.” Under RL or CSR independence, between row or column independence is violated for the *Q*-symmetry contingency table. For example, under RL with two classes, *Q*_1,*m*_ and *Q*_2,*m*_ are highly correlated; in fact, correlation between them is −1 when *k* = 2, since *Q*_2,*m*_ = *q*_*m*_ − *Q*_1,*m*_. Furthermore, *Q*_*i*,*m*_ and *Q*_*i*,*m*′_ are also highly dependent and so are *Q*_*i*,*m*_ and *Q*_*i*′,*m*′_. Hence, the suggested asymptotic distribution for *𝒳*_II_^2^ should be appropriate under sparse sampling only. However, our extensive Monte Carlo simulations suggest that the asymptotic approximation with the reduced contingency table using *χ*_2(*k*−1)_^2^ distribution seems to hold for completely mapped data as well. Therefore, the *test seems to be appropriate for both sparsely sampled or completely mapped data*. Yet, finding the exact and asymptotic distribution of *𝒳*_II_^2^ is still open problems.

## 3. Fisher's Exact Test for the *Q*-Symmetry Contingency Table

Fisher's exact test is widely used for contingency tables for small sample sizes (see, e.g., [17]). However, it can neither be used to test Pielou's first type of symmetry nor Dixon's symmetry test for two classes nor for their extensions to *k* > 2 case, since we only consider the equality of off-diagonal entries in these tests, while Fisher's exact test is used to detect any departure from independence for all cell count in the contingency table. An alternative exact test for small *n*_*t*_ = *N*_12_ + *N*_21_ can be obtained by using the usual binomial test for Pielou's first type of symmetry test under the appropriate sampling framework. The use of exact tests on NNCTs for testing segregation/association is discussed in Ceyhan [18]. We can apply Fisher's exact test for the 2 × 6  *Q*-symmetry contingency table given in Table 2 (or the reduced 2 × 3 contingency table) for Pielou's second type of symmetry test.

If calculated manually, Fisher's exact test is feasible only for small size contingency tables. Furthermore, the underlying assumption of the Fisher's exact test is that the total number of observations, row and column sums are fixed, so Fisher's exact test is a *test conditional on the marginals*. For *k* × *l* contingency tables, when *k* = *l* = 2, then Fisher's exact test can be one-sided or two-sided, whereas, when min⁡(*k*, *l*) > 2 (hence for the *Q*-symmetry contingency table), it is two-sided only [17].

There are numerous ways to obtain *p* values for the two-sided alternatives for exact inference on contingency tables [17]. These *variants of Fisher's exact test* are described below. The *p* values based on Fisher's exact tests tend to be more conservative than most approximate (asymptotic) ones [17].

### 3.1. Variants of Fisher's Exact Test for Two-Sided Alternatives

To find the *p* values for Fisher's exact test, we find the probabilities of the contingency tables obtained from the distribution with the same row and column marginal sums. For the two-sided alternatives, a recommended method is adding up probabilities of contingency tables of the same size and smaller than the probability associated with the current table. Alternatively, twice the one-sided *p* value can also be used for a 2 × 2 contingency table [17]. Let the probability of the *k* × *l* contingency table, *C*_*T*_, be denoted as *f*(*C*_*T*_), where min⁡(*k*, *l*) > 2, and let sum of row *i* be *r*_*i*_, let sum of column *j* be *c*_*j*_, and let entry *i*, *j* be *N*_*ij*_. Then the probability of the contingency table, *C*_*T*_, is [13]

(22) $$\begin{array}{c} f\left(C_{T}\right)=\frac{\prod_{i=1}^{k}\left(r_{i}!/\left(N_{i1}!N_{i2}!\ldots N_{il}!\right)\right)}{n!/\left(c_{1}!c_{2}!\ldots c_{l}!\right)}. \end{array}$$

In particular, for the 2 × 3 reduced *Q*-symmetry contingency table, we get

(23) $$\begin{array}{c} f\left(C_{T}\right)=\frac{\left(n_{1}!/\left(Q_{1,0}!Q_{1,1}!Q_{1,2}!\right)\right)\left(n_{2}!/\left(Q_{2,0}!Q_{2,1}!Q_{2,2}!\right)\right)}{n!/\left(Q_{0}!Q_{1}!Q_{2}!\right)}. \end{array}$$

Let the probability of the current contingency table be denoted as *p*_*t*_.

For summing the *p* values of more extreme tables than the current table in both directions, the following variants of the exact test are obtained. The *p* value is calculated as *p* = ∑_*S*_ *f*(*C*_*T*_) for the appropriate choice of the set of contingency tables, *S*, as follows:

- *table-inclusive version*, denoted as *p*_inc_: take *S* = {*C*_*T*_ : *f*(*C*_*T*_) ≤ *p*_*t*_};
- *twice-table-inclusive version*, *p*_*t*,inc_: the probability of the observed table is included twice, once for each side;
- *table-exclusive version*, *p*_exc_: table-inclusive minus *p*_*t*_;
- *mid-p version*, *p*_mid_: table-exclusive plus one-half the *p*_*t*_;
- *Tocher corrected version*, *p*_Toc_, is obtained as follows.

Tocher's correction makes Fisher's exact test less conservative, by including the probability for the current table based on a randomized test [19]. When table-inclusive version of the *p* value, *p*_inc_, is larger than the level of the test *α*, but table-exclusive version, *p*_exc_, is less than *α*, a random number, *U*, is generated from uniform distribution in (0,1), and if *U* ≥ (*α* − *p*_exc_)/*p*_*t*_, then *p*_inc_ is used as the *p* value, otherwise *p*_exc_ is used as the *p* value. That is,

(24) $$\begin{array}{c} p_{\text{Toc}}=\{\begin{array}{cc} p_{\text{inc}} & \text{if}\;U\geq \frac{\left(\alpha -p_{\text{exc}}\right)}{p_{t}},\\ p_{\text{exc}} & \text{otherwise}. \end{array} \end{array}$$

Observe that *p*_exc_ = *p*_inc_ − *p*_*t*_ and *p*_mid_ = *p*_exc_ + *p*_*t*_/2. Additionally, *p*_exc_ ≤ *p*_Toc_ ≤ *p*_inc_ < *p*_*t*,inc_ and *p*_exc_ < *p*_mid_ < *p*_inc_ < *p*_*t*,inc_.

## 4. Asymptotic Power Analysis

The null hypotheses are different for the symmetry tests and so are the alternative hypotheses. This makes the comparison of the tests inappropriate even for large samples; however, under specific alternatives and assumptions, we can estimate asymptotic efficiency scores, such as those of Pitman asymptotic efficiency. A reasonable test should have more power as the sample size increases. So, we first prove the consistency of the tests in question under appropriate hypotheses.

### 4.1. Consistency of Tests

The consistency of Pielou's second and first types of symmetry tests is shown as below.

**Theorem**

For properly sparsely sampled data under the row-wise multinomial framework, Pielou's second type of symmetry test for the multiclass case with the *k* × 6 contingency table; that is, the test rejecting $H_{o}:{\vec{p}}_{i}=(p_{0},\ldots ,p_{5})$ for all *i* ∈ {1,2,…, *k*} for *𝒳*_*II*_^2^ > *χ*_5(*k*−1)_^2^(1 − *α*) with *𝒳*_*II*_^2^ = ∑_*m*=0_^5^∑_*i*=1_^*k*^((*Q*_*i*,*m*_ − *N*_*i*_ *Q*_*m*_/*n*)^2^/(*N*_*i*_ *Q*_*m*_/*n*)) is consistent.

**Proof**

In the multiclass case with *k* ≥ 2, deviations from *H*_*o*_ may have many possible forms. In any deviation from *H*_*o*_, that is, under *H*_*a*_, for large *n*, *𝒳*_II_^2^ is approximately distributed as a *χ* ^2^ distribution with noncentrality parameter $\lambda (\vec{ɛ})$ and 5(*k* − 1) df, which is denoted as $\chi_{5(k-1)}^{2}(\lambda (\vec{ɛ}))$. The noncentrality parameter is a quadratic form which can be written as $\vec{\mu }{\left(\vec{ɛ}\right)}^{\prime }A\vec{\mu }(\vec{ɛ})$ for some positive definite matrix *A* of rank 5(*k* − 1) (see, e.g., [20]); hence, $\lambda (\vec{ɛ})>0$ under *H*_*a*_. Then, for large *n*, the null and alternative hypotheses are equivalent to *H*_*o*_ : *λ* = 0 versus $H_{a}:\lambda =\lambda (\vec{ɛ})>0$. Then, by standard arguments for the consistency of *χ* ^2^-tests, the result follows.

The consistency of Pielou's second type of symmetry test for the 2 × 3 (reduced) contingency table can be shown similarly.

**Theorem**

Let the NNCT be constructed by a random sample of base-NN pairs (i.e., data is obtained by an appropriate sparse sampling) under an overall multinomial framework. Then, Pielou's first type of symmetry test, that is, the test rejecting *H*_*o*_ : **E**[*N*_*ij*_] = **E**[*N*_*ji*_] for all *i*, *j* with *i* ≠ *j* against *H*_*a*_ : **E**[*N*_*ij*_] ≠ **E**[*N*_*ji*_] for some *i*, *j* with *i* ≠ *j* for *𝒳*_*I*_^2^ > *χ*_*k*(*k*−1)/2_^2^(1 − *α*) with *𝒳*_*I*_^2^ as in (4) is consistent. The corresponding one-sided tests using *Z*_*I*_ given in (3) are also consistent.

**Proof**

In the two-class case, recall that this test is the same as McNemar's test with a continuity correction. Given that *N*_12_ + *N*_21_ = *n*_*t*_, the correction is used for small *n*_*t*_ and its impact vanishes as *n*_*t*_ → *∞*. So we prove the consistency for the uncorrected version (i.e., for the test without continuity correction), *𝒳*_I_^2^ = (*N*_12_ − *N*_21_)^2^/(*N*_12_ + *N*_21_). Let *T*_*n*_*t*__ = *N*_12_/*n*_*t*_ − 1/2. Then, under *H*_*o*_, we have *N*_12_ ~ BIN(*n*_*t*_, 1/2). So $Z_{\text{I}}=\left(N_{12}-n_{t}/2\right)/\sqrt{n_{t}/4}=T_{n_{t}}/\sqrt{1/(4\;n_{t})}=\left(N_{12}-N_{21}\right)/\sqrt{N_{12}+N_{21}}$. Hence, *Z*_I_ is approximately distributed as *N*(0,1) for large *n*_*t*_ under the null hypothesis and a normal distribution under alternative hypothesis. Notice that *Z*_I_^2^ = *𝒳*_I_^2^ in the uncorrected version. Under *H*_*o*_, **E**[*T*_*n*_*t*__] = 0 and, under *H*_*a*_, **E**[*T*_*n*_*t*__ | *H*_*a*_] = *ɛ* > 0 or **E**[*T*_*n*_*t*__ | *H*_*a*_] = *ɛ* < 0. Then, by the standard arguments for the consistency of *z*-tests, the test using *Z*_I_ is consistent. The *α*-level test based on *𝒳*_I_^2^ is equivalent to *α*-level two-sided test based on *Z*_I_. Hence, the consistency of *𝒳*_I_^2^ follows as well. For *k* > 2, consistency of $Z_{\text{I}}^{ij}=\left(N_{ij}-N_{ji}\right)/\sqrt{N_{ij}+N_{ji}}$ is similar to *Z*_I_ with (*i*, *j*) = (1,2) and consistency of *𝒳*_I_^2^ follows as in the proof of Theorem 2.

**Theorem 4**

Let the NNCT be constructed from a completely mapped data under RL. Then Dixon's symmetry test, that is, the test rejecting *H*_*o*_ : **E**[*N*_*ij*_] = **E**[*N*_*ji*_] for all *i*, *j* with *i* ≠ *j* against *H*_*a*_ : **E**[*N*_*ij*_] ≠ **E**[*N*_*ji*_] for some *i*, *j* with *i* ≠ *j* for *𝒳*_*D*_^2^ > *χ*_*k*(*k*−1)/2_^2^(1 − *α*) with *𝒳*_*D*_^2^ as in (18) is consistent. The corresponding one-sided tests using *Z*_*D*_ given in (10) are also consistent.

**Proof**

In the two-class case, let $T_{D}=\left(N_{12}/n-N_{21}/n\right)/\sqrt{Var[N_{12}/n+N_{21}/n]}$; then *T*_*D*_ = *Z*_*D*_. Under RL, **E**[*Z*_*D*_] = 0 since **E**[*N*_12_] = **E**  [*N*_21_] and *Z*_*D*_ is approximately distributed as *N*(0,1) for large *n*_*i*_ under the null hypotheses. Under *H*_*a*_, **E**[*Z*_*D*_ | *H*_*a*_] = *ɛ* > 0 or **E**[*Z*_*D*_ | *H*_*a*_] = *ɛ* < 0 and **V** **a** **r**[*N*_*ij*_/*n*] = *p*_*ij*_(*ɛ*)/*n* + *Qp*_*iij*_(*ɛ*)/*n* ^2^ + (1 − 3/*n* − *Q*/*n* ^2^)*p*_*iij*_(*ɛ*)−(*p*_*ij*_(*ɛ*))^2^ and **C** **o** **v**[*N*_*ij*_/*n*, *N*_*ji*_/*n*] = *Rp*_*ij*_(*ɛ*)/*n* ^2^ + (1/*n* − *R*/*n* ^2^)(*p*_*iij*_(*ɛ*) + *p*_*jji*_(*ɛ*))+(1 − 3/*n* − *Q*/*n* ^2^ + *R*/*n* ^2^)*p*_*ijjj*_(*ɛ*) − *p*_*ij*_(*ɛ*)*p*_*ji*_(*ɛ*). So, under *H*_*a*_, **V** **a** **r**[*N*_*ij*_] → 0 and **C** **o** **v**[*N*_*ij*_, *N*_*ji*_] → 0 as *n*_*i*_ → *∞*. Hence, the test using *Z*_*D*_ is consistent. The *α*-level test based on *𝒳*_*D*_^2^ is consistent as in the proof of Theorem 2, since *𝒳*_*D*_^2^ is a quadratic based on *Z*_*D*_^*ij*^ values; that is, *𝒳*_*D*_^2^ ~ *χ*_df_^2^(*λ*(*ɛ*)) for some *λ*(*ɛ*) > 0.

**Remark 5**

The consistency result for Pielou's first type of symmetry test is only for sparsely sampled data with contingency table from the overall multinomial framework. Pielou's second type of symmetry test is consistent only for sparsely sampled data with the row-wise multinomial framework. For completely mapped data, these tests do not have the appropriate size. In particular, Monte Carlo simulations suggest that Pielou's first type of symmetry test (with *χ* ^2^ approximation or exact binomial version) is extremely conservative. See also Section 5.

### 4.2. Asymptotic Power Comparison of the Tests

The power of a test in hypothesis testing depends on the statistic being employed, sample size, the level of the test *α*, and the parameter(s) under *H*_*a*_. To be able to compare the tests, we should consider the asymptotics with only *n* → *∞*, where the asymptotic power tends to 1 for consistent tests. Since the power depends on multiple parameters, many asymptotic efficiency methods are introduced to compare asymptotic power performance. See [21] for a brief survey of asymptotic efficiency measures.

The tests with small level and high power under alternatives close to null hypothesis have practical importance. Hence, Pitman asymptotic efficiency (PAE) is widely used in practice. PAE analysis provides for an investigation of local power around *H*_*o*_, which involves the limit as *n* → *∞* together with the limit of alternative parameter converging to the null parameter. See, for example [22, 23] for more details.

**Remark**

Suppose that the distribution *F* under consideration can be indexed by Θ⊆ℝ and consider *H*_*o*_ : *θ* = *θ*_0_ versus *H*_*a*_ : *θ* > *θ*_0_. If the test statistic satisfies central limit theorem together with the Pitman's conditions [23] with *μ*_*n*_ = **E**[*T*_*n*_] and *σ*_*n*_^2^ = **V** **a** **r**[*T*_*n*_], then PAE of *T*_*n*_ is given by

(25) $$\begin{array}{c} \text{PAE}\left(T_{n}\right)=\underset{n\to \infty }{\lim}\frac{\mu_{n}^{2}\left(\theta =\theta_{0}\right)}{n\sigma_{n}^{2}}. \end{array}$$

If a test statistic, *T*_*n*_, converges in law to *χ*_*ν*_^2^ distribution as *n* → *∞*, then the local power approximation using asymptotic normality of *T*_*n*_ is not appropriate [24]. By suitable transformations, the corresponding test asymptotically boils down to *H*_*o*_ : *λ* = 0 versus *H*_*a*_ : *λ* > 0, where *λ* is the noncentrality parameter for the *χ*_*ν*_^2^ distribution. Therefore, we investigate the local power around *λ* = 0. Let *f*_*ν*_(*x*, *λ*) and *F*_*ν*_(*x*, *λ*) be the pdf and cdf of *χ*_*ν*_^2^(*λ*) distribution, respectively.

**Theorem**

Suppose that *C* ^2^ is a test statistic which converges in law to *χ*_*ν*_^2^(*λ*) with *λ* = 0 under *H*_*o*_ and to *χ*_*ν*_^2^(*λ*) with *λ* > 0 under *H*_*a*_. Then the local power for small *λ* (*λ* around 0) is given by

(26) β(λ,ν,α) ≈α+λ  (−α2+12[1−Fν+2(λ=0,χν2(0,1−α))]).

The proof is provided in the Appendix.

#### 4.2.1. Asymptotic Local Power Analysis of the Tests

Pielou's first type of symmetry test is used for testing *H*_*o*_ : **E**[*N*_12_] = **E**[*N*_21_] versus *H*_*a*_ : **E**[*N*_12_] ≠ **E**[*N*_21_]. Under *H*_*o*_, given that *N*_12_ + *N*_21_ = *n*_*t*_, *N*_12_ ~ BIN(*n*_*t*_, 1/2), since **E**[*N*_12_]/(**E**[*N*_12_] + **E**[*N*_21_]) = 1/2. Under *H*_*a*_, *N*_12_ ~ BIN(*n*_*t*_, 1/2 + *ɛ*_1_) for *ɛ*_1_ ∈ (0,1/2). Let *T*_*n*_*t*__ = *N*_12_/*n*_*t*_ − 1/2. Then *T*_*n*_*t*__^2^ = (*N*_12_ − *N*_21_)^2^/*n*_*t*_ which is equal to *𝒳*_I_^2^ (without Yates' correction) in (2). Under *H*_*o*_, **E**[*T*_*n*_*t*__] = 0 and **V** **a** **r**[*T*_*n*_*t*__] = 1/4*n*_*t*_ and under *H*_*a*_, let **E**[*T*_*n*_*t*__ | *H*_*a*_] = *ɛ*_1_. Next, let *μ*_*n*_*t*__ = **E**[*T*_*n*_*t*__] and *σ*_*n*_*t*__^2^(*T*_*n*_*t*__) = **V** **a** **r**[*T*_*n*_*t*__]. Then *μ*_*n*_*t*__ and *σ*_*n*_*t*__ satisfy the Pitman conditions and *μ*_*n*_*t*__′(*ɛ*_1_ = 0) = 1 (see [23]). Then by Remark 6, the PAE of *T*_*n*_*t*__ (for the parameterization *H*_*a*_ : **E**[*N*_12_] − **E**[*N*_21_] = *ɛ*_1_) is

(27) $$\begin{array}{c} \text{PAE}\left(T_{n_{t}}\right)=\text{PAE}\left(Z_{\text{I}}\right)=4. \end{array}$$

The asymptotic local power for Dixon's symmetry test for the two-class case can also be investigated with PAE analysis. For Dixon's symmetry test for the two-class case, consider *T*_*n*_ = (*N*_12_ − *N*_21_)/*n*. Then

(28) ZD=TnVar[Tn]=N12−N21Var[N12]+Var[N21]−2Cov[N12,N21].

Let *μ*_*n*_ = **E**[*T*_*n*_], *σ*_*n*_^2^ = **V** **a** **r**[*T*_*n*_], *p*_*q*_ = **E**[*Q*/*n*], and *p*_*r*_ = **E**[*R*/*n*]. That is, *p*_*q*_ is the probability of a point being a shared NN and *p*_*r*_ is the probability of a pair being reflexive. Then, under *H*_*o*_, **E**[*T*_*n*_ | *H*_*o*_] = 0 and

(29) Var[Tn ∣ Ho] =(Var[N12]+Var[N21]−2Cov[N12,N21])n2,

where for large *n*_*i*_

(30) $$\begin{array}{c} Var\left[N_{ij}\right]\approx \nu_{i}\nu_{j}\left[1+p_{q}\nu_{i}-\left(3+p_{q}-p_{r}\right)\nu_{i}\nu_{j}\right] \end{array}$$

for (*i*, *j*)∈{(1,2), (2,1)} and

(31) Cov[N12,N21] ≈ν1ν2[pr+(1−pr)(ν1+ν2)−(3+pq−pr)ν1ν2],

and, under *H*_*a*_, let **E**[*T*_*n*_ | *H*_*a*_] = *ɛ*_2_. Then, by Remark 6, PAE of *Z*_*D*_ (for the parameterization *H*_*a*_ : **E**[(*N*_12_ − *N*_21_)/2] = *ɛ*_2_) is given by

(32) PAE(ZD)=1Var[N12]+Var[N21]−2Cov[N12,N21]=1ν1(1−ν1)pq.

For the asymptotic relative efficiency between Pielou's first type of symmetry test and Dixon's symmetry test to make sense, the null assumptions for these tests should match and so should the alternatives and the parameterizations of the alternatives (under which PAE scores are computed). Otherwise, PAE(*Z*_I_) and PAE(*Z*_*D*_) would not be comparable. In particular, since the (appropriate) null and alternatives are different for these tests, we refrain from computing asymptotic relative efficiency for these tests. On the other hand, for Dixon's symmetry test with varying *ν*_1_ and *p*_*q*_, notice that PAE(*Z*_*D*_) increases as *ν*_1_ gets closer to 0 or 1 or *p*_*q*_ gets smaller. For example, for fixed *p*_*q*_, PAE of *Z*_*D*_ gets larger as the relative abundances of the classes get more and more different (which implies that *ν*_1_ gets closer to 0 or 1). The smallest PAE(*Z*_*D*_) values are obtained when *ν*_1_ = *ν*_2_ = 1/2 for any *p*_*q*_ > 0. That is, the power of Dixon's test for spatial symmetry (in mixed NN structure) highly depends on the relative abundances of the classes. The PAE of *Z*_*D*_^*ij*^ in the multiclass case is similar.

## 5. Empirical Performance of the Tests

In this section we investigate the finite sample behavior of the tests under various patterns via Monte Carlo simulations.

### 5.1. Empirical Performance Analysis under RL and CSR Independence

Both CSR independence and RL patterns imply symmetry in the mixed or shared NN structure. That is, under these cases, the asymmetry would occur at expected levels. More specifically, we expect that **E**[*N*_12_] = **E**[*N*_21_] = *n*_1_ *n*_2_/*n* would hold for symmetry in mixed NN structure, and ${\vec{p}}_{1}={\vec{p}}_{2}$ in (19) would hold for symmetry in shared NN structure. Hence, these patterns imply our null hypotheses and hence can be used to assess the empirical size performance of the tests.

In what follows empirical size estimates are based on the asymptotic critical values (except for the exact tests). In particular, for a test, *T*, with a *χ*_df_^2^ distribution asymptotically, empirical sizes are estimated as follows. Let *T*_*i*_ be the value of test statistic for the sample generated at *i*th Monte Carlo replication for *i* = 1,2,…, *N*_mc_. Then the empirical size of *T* at level *α* = 0.05, denoted ${\hat{\alpha }}_{T}$, is computed as ${\hat{\alpha }}_{T}=(1/N_{\text{mc}})\sum_{i=1}^{N_{\text{mc}}}I$  (*T*_*i*_ > *χ*_df_^2^(0.95)), where *χ*_df_^2^(0.95) is the 95th percentile of *χ*_df_^2^ distribution. For an exact test, let *p*_*i*_ be the *p* value for *i*th sample generated. Then the empirical size of this test, denoted ${\hat{\alpha }}_{E}$, is computed as ${\hat{\alpha }}_{E}=(1/N_{\text{mc}})\sum_{i=1}^{N_{\text{mc}}}I$  (*p*_*i*_ < 0.05). With *N*_mc_ = 10000, an empirical size estimate larger than 0.0536 is deemed liberal, while an estimate smaller than 0.0464 is deemed conservative at .05 level (based on binomial critical values with *n* = 10000 trials and probability of success 0.05).

#### 5.1.1. Empirical Size Analysis under CSR Independence

We consider the two-class case, with classes 1 and 2 (also referred to as the classes *X* and *Y*, resp.) of sizes *n*_1_ and *n*_2_, respectively. Let {*X*_1_,…, *X*_*n*_1__} be the set of class 1 points and let {*Y*_1_,…, *Y*_*n*_2__} be the set of class 2 points. Under *H*_*o*_, at each of *N*_mc_ = 10000 replicates, we generate *X* and *Y* points independently of each other and iid from *𝒰*((0,1)×(0,1)), the uniform distribution on the unit square. We consider two cases for CSR independence.

*Case  1*. We generate *n*_1_ = *n*_2_ = *n* = 10,20,30,40,50 points iid from *𝒰*((0,1)×(0,1)). In this case, the sample sizes are equal and increasing.

*Case  2*. To determine the influence of differences in the sample sizes (i.e., differences in relative abundances) on the empirical levels of the tests, we generate the samples from the CSR independence pattern with *n*_1_ = 20 and *n*_2_ = 20,30,…, 60.

The empirical significance levels (under CSR independence Cases 1 and 2) for the symmetry tests are presented in Table 3, where ${\hat{\alpha }}_{\text{I}}^{P}$ and ${\hat{\alpha }}_{\text{I}}^{P^{\prime }}$ are the (estimated) empirical significance levels for Pielou's first type of symmetry test using *χ* ^2^ approximation with and without Yates' continuity correction, respectively; ${\hat{\alpha }}_{\text{bin}}^{P}$ is for the exact binomial version of Pielou's first type of symmetry test conditional on *N*_12_ + *N*_21_ = *n*_*t*_; ${\hat{\alpha }}_{\text{II}}^{P}$ is the empirical significance level for Pielou's second type of symmetry test; ${\hat{\alpha }}_{S}^{D}$ is for Dixon's symmetry test. Notice that Pielou's first type of symmetry tests and the exact binomial test are extremely conservative. Furthermore, we recommend the use of the Monte Carlo randomized versions of these tests or with Monte Carlo critical values rather than the approximate asymptotic critical values. A Monte critical value is determined as the appropriately ranked value of the test statistic in a certain number of generated data sets under the null hypothesis. The other tests seem to be of the desired level for each sample size considered.

The empirical significance levels for the exact tests on the *Q*-symmetry contingency table under CSR independence Cases 1 and 2 are presented in Table 4, where ${\hat{\alpha }}_{\text{inc}}$ is the empirical significance level for the two-sided test with the table-inclusive version, ${\hat{\alpha }}_{\text{exc}}$ is for table-exclusive version, ${\hat{\alpha }}_{\text{mid}}$ is for mid-*p* value version, and ${\hat{\alpha }}_{\text{Toc}}$ is for Tocher corrected version. Notice that only the table exclusive version is about the desired level, while the others are more conservative. Hence, in what follows, only the table exclusive version will be employed for exact inference on *Q*-symmetry contingency table.

#### 5.1.2. Empirical Size Analysis under RL

For the RL pattern, the locations of the points are given and the marks or class labels are assigned randomly to these points. The pattern generating these locations is referred to as the *background pattern* henceforth. Let *𝒵*_*n*_ = {*Z*_1_, *Z*_2_,…, *Z*_*n*_} be the given set of locations for *n* points from the background pattern. We consider RL of class labels of 1 and 2 (or *X* and *Y*) to these points which are generated from homogeneous or clustered patterns. We generate 100 different realizations of the background pattern, *𝒵*_*n*_, to mitigate the influence of a particular background realization on the size performance of the tests. At each background realization, *n*_1_ of the points are labeled as class 1 and the remaining *n*_2_ = *n* − *n*_1_ points are labeled as class 2. 

*Types of the Background Patterns*

*Case  1*. The background points, *𝒵*_*n*_, are generated iid in the unit square (0,1) × (0,1). That is, $Z_{i}\overset{\text{iid}}{\sim }𝒰((0,1)\;\times \;(0,1))$ for *i* = 1,2,…, *n*. To determine the effect of increasing equal sample sizes, we consider *n*_1_ = *n*_2_ = *n* = 10,20,…, 50. The above RL scheme is repeated 1000 times for each (*n*_1_, *n*_2_) combination of background realization. 

*Case  2*. The background points, *𝒵*_*n*_, are generated as in Case 1 above with *n*_1_ = 20 and *n*_2_ = 20,30,…, 60 to determine the differences in the sample sizes with number of class 1 points fixed and number of class 2 points increasing. The above RL scheme is repeated 1000 times for each (*n*_1_, *n*_2_) combination of background realization. 

*Case  3*. We generate the background points from a Matérn cluster process. More specifically, *Z*_*i*_ points are generated from MatClust(*κ*, *r*, *μ*) process, which is the Matérn cluster process in the unit square [25]. In this process, first “parent” points are generated from a Poisson process with intensity *κ* and then one replaces each parent point by *N* new points which are generated iid inside the circle centered at the parent point with radius *r*. Here *N* is also random; *N* ~ Poisson(*μ*). At each background realization, one realization of *𝒵*_*n*_ is generated from MatClust(*κ*, *r*, *μ*). Let *n* be the number of points in a particular realization. Then *n*_1_ = ⌊*n*/2⌋ of these points are labeled as class 1, where ⌊*x*⌋ stands for the floor of *x* and *n*_2_ = *n* − *n*_1_ as class 2. In our simulations, we use *κ* = 2,4,…, 10, *μ* = ⌊100/*κ*⌋, and *r* = 0.1. That is, we take (*κ*, *μ*)∈{(2,50), (4,25)…, (10,10)}, in order to have about 100 *Z* points, where about half of them are class 1 and the other half are class 2 points on the average.

In RL Cases 1 and 2, the points are from HPP in the unit square (with fixed *n*_1_ and *n*_2_), where Case 1 is for assessing the effect of increasing but equal sample sizes on the tests, while Case 2 is for assessing the effect of increasing differences in relative abundances of the classes (with one class size being fixed, while the other is increasing). On the other hand, in Case 3, we have the background realizations with cluster centers and cluster numbers being random. On the average, with increasing *κ*, the number of clusters tend to increase, and cluster sizes tend to decrease (so as to have fixed class sizes on the average). Hence, in Case 3, we investigate the influence of increasing number of clusters with randomly determined centers on the size performance of the tests.

The empirical size estimates of the tests under RL Cases 1–3 are presented in Table 5. The empirical size performance of the tests under Cases 1 and 2 is similar to that under CSR independence Cases 1 and 2, respectively. Tests of Pielou's first type of symmetry are extremely conservative, while the other tests are about the desired level. The empirical size estimates of the exact test for Pielou's second type of symmetry (the table exclusive version) are denoted as ${\hat{\alpha }}_{\text{II}}^{F}$ for notational convenience. Furthermore, ${\hat{\alpha }}_{\text{II}}^{F}$ is close to the nominal level for all sample sizes or *κ* values. Notice also that the size estimates of the tests are not influenced by the number of clusters, *κ*, when the class sizes are fixed.

Based on the empirical size performance of the tests, we observe that variants of Pielou's first type of symmetry test are extremely conservative and hence are not reliable in practice. On the other hand, Pielou's second type of symmetry test and Dixon's symmetry test are appropriate for balanced or unbalanced sample sizes. When the relative abundances of the classes are close to one (i.e., *n*_*i*_/*n*_*j*_ ≈ 1 for *i* ≠ *j*), we call the class sizes to be balanced, but when the relative abundances deviate substantially from one we call the class sizes to be unbalanced. For the exact tests on *Q*-symmetry contingency table, we recommend the table-exclusive version.

### 5.2. Empirical Performance of the Tests under Various Other Patterns

To assess the empirical performance of the tests, we consider six pattern cases for the NN structure. Empirical rejection rate estimates are computed as the size estimates in Section 5.1. 

*Case I*. For the first class of patterns, we generate $X_{i}\overset{\text{iid}}{\sim }𝒰((0,1)\times (0,1))$ for *i* = 1,…, *n*_1_ and $Y_{j}\overset{\text{iid}}{\sim }\text{BVN}(1/2,1/2,\sigma_{1},\sigma_{2},\rho )$ for *j* = 1,…, *n*_2_, where *BVN*(*μ*_1_, *μ*_2_, *σ*_1_, *σ*_2_, *ρ*) is the bivariate normal distribution with mean (*μ*_1_, *μ*_2_) and covariance $\left[\begin{array}{cc} \sigma_{1} & \rho \\ \rho & \sigma_{2} \end{array}\right]$. In our simulations, we set *σ*_1_ = *σ*_2_ = *σ* and *ρ* = 0. We consider three patterns in which

(33) $$\begin{array}{c} \left(\text{i}\right)\text{:}\;\;\;\sigma =\frac{1}{10},\;\;\left(\text{ii}\right)\text{:}\;\;\;\sigma =\frac{1}{20},\;\;\left(\text{iii}\right)\text{:}\;\;\;\sigma =\frac{1}{30}. \end{array}$$

The classes 1 and 2 (i.e., *X* and *Y*) have different distributions with different local intensities. In particular, *X* points constitute a realization of HPP process in the unit square, while *Y* points are clustered around the center of the unit square, namely (1/2,1/2). In fact, the level of clustering of *Y* points increases as *σ* decreases.

The means (±SD (standard deviations)) of the off-diagonal entries, *N*_12_, *N*_21_, and their difference *N*_12_ − *N*_21_ and empirical rejection rate estimates under the patterns, (i), (ii), and (iii), with *n*_1_ = *n*_2_ = 40 are presented in Table 6(a), where ${\hat{\beta }}_{\text{I}}^{P}$ and ${\hat{\beta }}_{\text{I}}^{P^{\prime }}$ stand for the empirical rejection rates for Pielou's first type of symmetry test using *χ* ^2^ approximation with and without Yates' continuity correction, respectively; ${\hat{\beta }}_{\text{bin}}^{P}$ is for the exact binomial version of Pielou's first type of symmetry test conditional on *N*_12_ + *N*_21_ = *n*_*t*_; ${\hat{\beta }}_{S}^{D}$ is for Dixon's symmetry test; ${\hat{\beta }}_{\text{II}}^{P}$ is for Pielou's second type of symmetry test; ${\hat{\beta }}_{\text{II}}^{F}$ is for the exact test on the *Q*-symmetry contingency table. Notice that, under Case I patterns, the off-diagonal entries, *N*_12_, *N*_21_, in the NNCTs tend to be much smaller than expected under *H*_*o*_ : **E**[*N*_12_] = **E**[*N*_21_] = *n*_1_ *n*_2_/*n* = 20 and *N*_12_ values tend to be larger than *N*_21_ values which suggests asymmetry in the mixed NN structure. Furthermore, *N*_12_, *N*_21_ tend to decrease with decreasing *σ*. That is, when the level of clustering of *Y* points in the center of the unit square increases (i.e., level of segregation of *Y* points from *X* points increases), the off-diagonal entries tend to decrease (in a similar fashion). The exact binomial version of Pielou's first type of symmetry test has the highest rejection rates which are increasing as *σ* is decreasing. The rejection rate estimates for all other symmetry tests are significantly smaller than the nominal level of .05, indicating lack of asymmetry in the mixed and shared NN structure. However, the fact that off-diagonal entries are small seems to render the asymptotic approximations inappropriate. Although the difference of the off-diagonal entries is larger than zero, the standard deviations of the differences are much smaller compared to those under CSR independence or RL (see also Table 7). Moreover, the exact binomial test is not appropriate either due to the dependence between trials (hence dependence between rows of the NNCT) for spatial data. Thus, in this situation, we recommend performing Monte Carlo randomization to determine more reliable rejection rate estimates. To that end, for each of the 100 generated samples under each of Case I patterns, 1000 Monte Carlo resampling is performed, and rejection rate for a test is estimated based on how many of the test statistics on resamplings are at and above the original test statistic. The corresponding Monte Carlo randomization rejection rate estimates are presented in Table 6(b), where the binomial version of Pielou's first type of symmetry test is omitted since it is conditional on *N*_12_ + *N*_21_ = *n*_*t*_ which is not fixed under Monte Carlo randomization steps. The rejection rate estimates are high for all tests and much higher than the nominal rate of 0.05. Hence, Case I patterns are actually providing significant asymmetry in mixed and shared NN structure, which was not revealed by the asymptotic approximation of the tests. Hence, this pattern is actually an alternative pattern for both symmetry structures, and the rejection rates are in fact power estimates under this alternative pattern. The highest power estimates are observed for Monte Carlo randomized version of Pielou's first type of test (and lowest estimates are for Dixon's symmetry test). Furthermore, the power estimates for Pielou's second type of symmetry tests are very similar. The power estimates for Monte Carlo randomized version of Pielou's first type of symmetry test and the two versions of Pielou's second type of symmetry test increase and those for other tests decrease as *σ* decreases. 

*Case II*. For Case II, we consider the following three patterns. First, we generate $X_{i}\overset{\text{iid}}{\sim }𝒰((0,1)\times (0,1))$ for *i* = 1,2,…, *n*_1_ and, for each *j* = 1,2,…, *n*_2_, we generate *Y*_*j*_ around a randomly picked *X*_*i*_ with probability *p* in such a way that *Y*_*j*_ = *X*_*i*_ + *R*_*j*_(cos⁡*T*_*j*_, sin*T*_*j*_)^*t*^, where *v* ^*t*^ represents transpose of the vector *v*, *R*_*j*_ ~ *𝒰*(0, min⁡_*i*≠*j*_ *d*(*X*_*i*_, *X*_*j*_)) and *T*_*j*_ ~ *𝒰*(0,2  *π*), or generate *Y*_*j*_ uniformly in the unit square with probability 1 − *p*. In the pattern generated, *Y*_*j*_ are more associated with *X*_*i*_. The three values of *p* constitute the following patterns:

(34) $$\begin{array}{c} \left(\text{i}\right)\text{:}\;\;p=.25,\;\;\left(\text{ii}\right)\text{:}\;\;p=.50,\;\;\left(\text{iii}\right)\text{:}\;\;p=.75. \end{array}$$

In this case, *X* points constitute a realization of a HPP process in the unit square, while *Y* points are clustered around the *X* points and the level of clustering increases as the parameter *p* increases. The means (±SD) of the off-diagonal entries, *N*_12_, *N*_21_, and their difference *N*_12_ − *N*_21_ and the empirical rejection rate estimates for Case II patterns with *n*_1_ = *n*_2_ = 40 are presented in Table 8. Notice that *N*_12_ and *N*_21_ in the NNCTs tend to be similar and larger than expected and *N*_12_ values tend to be slightly larger than *N*_21_ values. Furthermore, *N*_12_, *N*_21_ tend to increase with increasing *p*. That is, when the level of clustering of *Y* points around *X* points increases (i.e., level of association of *Y* points with *X* points increases), the off-diagonal entries tend to increase (in a similar fashion), indicating symmetry in the NN structure (but the difference between *N*_12_ and *N*_21_ values tends to increase with increasing *p*). Variants of Pielou's first type of symmetry test have virtually zero rejection rates, and, although Dixon's symmetry test has higher rejection rates than Pielou's first type, it has rates smaller than 0.05; hence there is symmetry in the mixed NN structure. In fact, under this pattern, expected value of the difference, *N*_12_ − *N*_21_, is mostly positive and with a larger variance compared to those under CSR independence and RL. However, there is severe asymmetry in shared NN structure, since Pielou's second type of symmetry test and its exact version have rejection rate estimates much larger than 0.05, and these estimates increase as *p* increases. Hence, this pattern type can serve as an alternative to symmetry in the shared NN structure and perhaps a null pattern for the tests of symmetry in the mixed NN structure for the range of *p* considered. However, using the asymptotic critical values based on the distribution under RL, the tests of symmetry in mixed NN structure would be extremely conservative for this null case. If the correct form of the variance and covariance terms can be determined as a function of *p*, then the tests for symmetry in mixed NN structure would have the desired level. Otherwise, Dixon's symmetry test and Pielou's first type of symmetry test can be used with Monte Carlo randomization. 

*Case III*. For the third class of patterns, we consider $X_{i}\overset{\text{iid}}{\sim }𝒰((0,1-s)\times (0,1-s))$ for *i* = 1,…, *n*_1_ and $Y_{j}\overset{\text{iid}}{\sim }𝒰((s,1)\times (s,1))$ for *j* = 1,…, *n*_2_. The three values of *s* constitute the following patterns:

(35) $$\begin{array}{c} \left(\text{i}\right)\text{:}\;\;s=\frac{1}{6},\;\;\left(\text{ii}\right)\text{:}\;\;s=\frac{1}{4},\;\;\left(\text{iii}\right)\text{:}\;\;s=\frac{1}{3}. \end{array}$$

Notice that these are the segregation patterns considered for Monte Carlo analysis in Ceyhan [12]. The means (±SD)) of the off-diagonal entries, *N*_12_, *N*_21_, and their difference *N*_12_ − *N*_21_ and the empirical rejection rate estimates for the segregation patterns are presented in Table 9. The off-diagonal entries, *N*_12_, *N*_21_, are very similar under these segregation patterns and are much smaller than expected under RL and tend to decrease as *s* (i.e., level of segregation) increases. Hence, mixed NN structure seems to be symmetric under these segregation patterns. The symmetry tests and the exact tests have very small rejection rates, with Pielou's first type and Dixon's symmetry tests having virtually zero rates and the others having rates lower than .05. There seems to be symmetry in both mixed and shared NN structure, since the null hypotheses seem to be satisfied. That is, the expected difference *N*_12_ − *N*_21_ is zero, and the cell counts in the *Q*-symmetry table are as expected under RL. However, the variances seem to be much smaller compared to the ones under RL or CSR independence (see Table 7). Thus, these segregation patterns can form null patterns for both types of symmetry tests; however, the correct variance and covariance terms should be computed; otherwise, the symmetry tests would be extremely conservative when the critical values are based on the distribution under RL or CSR independence. 

*Case IV*. We also consider patterns in which self-reflexive pairs are more frequent than expected by construction. We generate $X_{i}\overset{\text{iid}}{\sim }S_{1}$ for *i* = 1,…, ⌊*n*_1_/2⌋ and $Y_{j}\overset{\text{iid}}{\sim }S_{2}$ for *j* = 1,…, ⌊*n*_2_/2⌋. Then, for *k* = ⌊*n*_1_/2⌋ + 1,…, *n*_1_, we generate *X*_*k*_ = *X*_*k*−⌊*n*_1_/2⌋_ + *r*(cos⁡*T*_*j*_, sin*T*_*j*_)^*t*^ and, for *l* = ⌊*n*_2_/2⌋ + 1,…, *n*_2_, we generate *Y*_*l*_ = *Y*_*l*−⌊*n*_1_/2⌋_ + *r*(cos⁡*T*_*j*_, sin*T*_*j*_)^*t*^, where *r* ∈ (0,1) and *T*_*j*_ ~ *𝒰*(0,2  *π*). Appropriate small choices of *r* will yield an abundance of self-reflexive pairs. The three values of *r* we consider constitute the below self-reflexivity patterns at each support pair (*S*_1_, *S*_2_). Then the nine pattern combinations we consider are given by the following:

- *S*_1_ = *S*_2_ = (0,1)×(0,1), (a) *r* = 1/7, (b) *r* = 1/8, and (c) *r* = 1/9;
- *S*_1_ = (0,5/6)×(0,5/6) and *S*_2_ = (1/6,1)×(1/6,1), (a) *r* = 1/7, (b) *r* = 1/8, and (c) *r* = 1/9;
- *S*_1_ = (0,3/4)×(0,3/4) and *S*_2_ = (1/4,1)×(1/4,1) (a) *r* = 1/7, (b) *r* = 1/8, and (c) *r* = 1/9.

The means (±SD) of the off-diagonal entries, *N*_12_, *N*_21_, and their difference *N*_12_ − *N*_21_ and the empirical rejection rate estimates for Case IV patterns with *n*_1_ = *n*_2_ = 40 are presented in Table 10. In this case, the off-diagonal entries, *N*_12_, *N*_21_, tend to be very similar but smaller than expected under RL, indicating symmetry in mixed NN structure. Furthermore, as pattern changes from (i) to (iii) *N*_12_, *N*_21_ values tend to decrease, and, at each case IV pattern, *N*_12_, *N*_21_ values tend to decrease, as *r* (i.e., the level of self-reflexivity) decreases. Variants of Pielou's first type of symmetry test have small rejection rates (with the asymptotic versions having virtually zero rates and the exact version slightly higher rates); Dixon's symmetry test has rejection rates smaller than 0.05. Hence, we conclude that, under these self-reflexivity patterns, there is in fact symmetry in mixed NN structure, as the expected difference *N*_12_ − *N*_21_ is zero, but the variance of this difference is much smaller than that under RL. Hence, using the asymptotic distribution under RL, these tests would be extremely conservative. To get the desired level, one needs the correct form of the variances and covariances for Dixon's symmetry test under these patterns. On the other hand, Pielou's second type of symmetry tests has rejection rates about the nominal level of .05, indicating that these self-reflexivity patterns can also be viewed as the null pattern for symmetry in the shared NN structure. 

*Case V*. In this case, first, we generate $X_{i}\overset{\text{iid}}{\sim }𝒰((0,1)\times (0,1))$ and then generate *Y*_*j*_ as *Y*_*j*_ = *X*_*i*_ + *r*(cos⁡*T*_*j*_, sin*T*_*j*_)^*t*^, where *r* ∈ (0,1) and *T*_*j*_ ~ *𝒰*(0,2  *π*). In the pattern generated, appropriate choices of *r* will cause *Y*_*j*_ and *X*_*i*_ more associated. That is, a *Y* point is more likely to be the NN of an *X* point and vice versa. The four values of *r* we consider constitute the four association patterns:

(36) $$\begin{array}{c} \left(\text{i}\right)\text{:}\;\;r=\frac{1}{2},\;\;\left(\text{ii}\right)\text{:}\;\;r=\frac{1}{4},\;\;\left(\text{iii}\right)\text{:}\;\;r=\frac{1}{7},\\ \left(\text{iv}\right)\text{:}\;\;r=\frac{1}{10}. \end{array}$$

The patterns (i)–(iii) are also the association patterns considered for Monte Carlo analysis in Ceyhan [12].

The means (±SD) of the off-diagonal entries, *N*_12_, *N*_21_, and their difference *N*_12_ − *N*_21_ and the empirical rejection rate estimates for Case V patterns with *n*_1_ = *n*_2_ = 40 are presented in Table 11. Notice that the off-diagonal entries, *N*_12_, *N*_21_, tend to be at or above the expected value under RL and tend to increase as *r* (i.e., level of association) increases. Furthermore, *N*_12_ values tend to be slightly smaller than *N*_21_ values and the differences between *N*_12_ and *N*_21_ tend to decrease as *r* decreases. Variants of Pielou's first type of symmetry test have virtually zero rejection rates, and, under stronger association with 1/7 ≤ *r* ≤ 1/10, Dixon's symmetry test and exact and asymptotic versions of Pielou's second type of symmetry test have rates around .05, and, under moderate association with 1/2 ≤ *r* ≤ 1/4, these tests have rates mildly above .05. Hence, stronger association with 1/7 ≤ *r* ≤ 1/10 could serve as the null pattern for both types of symmetry tests, while, under moderate association with 1/2 ≤ *r* ≤ 1/4, the expected values are smaller in the negative direction compared to those under RL, with the variances about those under RL. 

*Case VI*. In this case, first, we generate $X_{i}\overset{\text{iid}}{\sim }𝒰((0,1)\times (0,1))$ for *i* = 1,2,…, *m*_1_ + *m*_2_ and, for each *X*_*i*_ generated, we find the distance of NN *X* point from *X*_*i*_, denoted *d*_*i*_^*x*^ (i.e., *d*_*i*_^*x*^ = min⁡_*i*≠*j*_ *d*(*X*_*i*_, *X*_*j*_)). Then we generate *Y*_*i*_ points as follows. First generate *R*_*i*_ from *𝒰*(0, *ρd*_*i*_^*x*^) and *θ*_*i*_ from *𝒰*(0,2*π*). Then set *Y*_*i*_ = *X*_*i*_ + *R*_*i*_(cos⁡(*θ*_*i*_), sin(*θ*_*i*_))^*t*^ for *i* = 1,2,…, *m*_1_. For *j* = 1,2,…, *m*_2_, we first generate *R*_*j*_ from *𝒰*(0, *ρd*_*j*_^*x*^) and *θ*_*j*_ from *𝒰*(0,2*π*). Then set *X*_*j*_′ = *X*_*i*_ + *R*_*j*_(cos⁡(*θ*_*j*_), sin(*θ*_*j*_))^*t*^ for *i* = *m*_1_ + 1, *m*_1_ + 2,…, *m*_1_ + *m*_2_ and *j* = 1,2,…, *m*_2_. Then we merge the *X*_*i*_'s and *X*_*j*_′'s to form the *X* points (which would have *n*_1_ = *m*_1_ + 2*m*_2_ many points). Moreover, we generate $Y_{j}^{\prime }\overset{\text{iid}}{\sim }𝒰((0,1)\times (0,1))$ for *j* = 1,2,…, *m*_2_. Let *d*_*j*_^*y*^ be the distance of NN *Y*′ point to *Y*_*j*_′ among the above generated *Y*′ points. For *k* = 1,2,…, *m*_2_, we first generate *R*_*k*_ from *𝒰*(0, *ρd*_*k*_^*y*^) and *θ*_*k*_ from *𝒰*(0,2*π*). Then set *Y*_*k*_′′ = *Y*_*j*_′ + *R*_*k*_(cos⁡(*θ*_*k*_), sin(*θ*_*k*_))^*t*^ for *k* = 1,2,…, *m*_2_. Then we merge the *Y*_*i*_'s, *Y*_*j*_′'s, and *Y*_*k*_′′'s to form the *Y* points (which would also have *n*_2_ = *m*_1_ + 2*m*_2_ many points). In the pattern generated, appropriate choices of *ρ* will cause *m*_1_ of the *X* points to have NNs more from *Y* points and *m*_2_ of the *X* points to have NNs more from *X* points; additionally, *m*_2_ of *Y* points would have NNs more from *Y* points. Hence, in this way, the off-diagonal entries (i.e., *N*_12_ and *N*_21_) would tend to be different, indicating asymmetry in mixed NN structure. The three values of *ρ* we consider constitute the following patterns:

(37) $$\begin{array}{c} \left(\text{i}\right)\text{:}\;\;\rho =\frac{1}{3},\;\;\left(\text{ii}\right)\text{:}\;\;\rho =\frac{2}{3},\;\;\left(\text{iii}\right)\text{:}\;\;\rho =1. \end{array}$$

The means (±SD) of the off-diagonal entries, *N*_12_, *N*_21_, and their difference *N*_12_ − *N*_21_ and the empirical rejection rate estimates for these patterns are presented in Table 12. The off-diagonal entries, *N*_12_, *N*_21_, tend to be different at or above the expected value under RL and they tend to increase as *ρ* increases. However, *N*_12_ values tend to be much smaller than *N*_21_ values, and their difference tends to decrease as *ρ* increases. The asymptotic versions of Pielou's first type of symmetry tests virtually have zero rejection rate, and the exact version has small rates which are slightly larger than .05 for *ρ* = 1/3. On the other hand, Dixon's test and versions of Pielou's second type of symmetry test have high rejection rates (much higher than 0.05), which decrease as *ρ* increases. Hence, there is strong asymmetry in mixed and shared NN structure, and the level of asymmetry is increasing with decreasing *ρ*. Thus, these patterns can serve as alternative patterns for both types of symmetry tests and the rejection rates are power estimates. Notice also that the asymmetry in the shared NN structure is stronger than the asymmetry in the mixed NN structure.

## 6. Pairwise versus One-versus-Rest Tests

In the multiclass case with *k* > 2, we first perform an overall omnibus test (as in ANOVA *F*-test for multigroup comparisons) and then, if the omnibus test is significant, then we perform post hoc tests to determine the specifics of the differences. These post hoc tests could be pairwise tests (as in pairwise *t*-tests) or one-versus-rest tests, where one class is compared with respect to all other classes combined. More specifically, with *k* > 2 classes, in the pairwise comparison, we only consider classes *i* ≠ *j*. The pairwise tests for Dixon's symmetry test and Pielou's second type of symmetry test can be defined in two different ways: (i) unrestricted pairwise symmetry tests and (ii) restricted pairwise symmetry tests. In the unrestricted version, for the pairwise test for classes *i*, *j*, *i* ≠ *j*, we keep all the points in consideration. That is, for Dixon's symmetry test, we extract *N*_*ij*_ and *N*_*ji*_ from the overall *k* × *k* NNCT and, in computing *Q* and *R* values, we do not ignore but use all the other classes. In the restricted version, we restrict our attention to two classes, *i*, *j*, with *i* ≠ *j*, only, and treat the classes as in the two-class case. That is, we only consider classes *i* and *j* and ignore the remaining classes and hence obtain a 2 × 2 NNCT just for classes *i* and *j* extract *N*_12_ and *N*_21_ from this NNCT, and compute *Q* and *R* for the data consisting of classes *i* and *j* only. The unrestricted version of Pielou's second type symmetry is based on the contingency table extracting only row *i* and *j* in the *Q*-symmetry contingency table. On the other hand, in the restricted version, we compute a 2 × 3  *Q*-symmetry contingency table based on data consisting of classes *i* and *j* only.

In the one-versus-rest type of test for class *i*, we pool the remaining classes and treat them as the other class in a two-class setting and hence the name *one-versus-rest test*. In a multiclass setting with *k* classes, there are *k* one-versus-rest type tests and $\left(\begin{array}{c} k\\ 2 \end{array}\right)=k(k-1)/2$ pairwise tests. As *k* increases, performing one-versus-rest analysis is computationally less intensive and easier to interpret.

Although Pielou's first type of symmetry test and Dixon's symmetry test were designed only for the two-class case, we have extended them to the multiclass case. Hence, if we have more than 2 classes; for Pielou's first type of symmetry, we can perform Bowker's test of symmetry in (4) (under the appropriate sampling distribution framework) as the overall test and use the test in (2) as the post hoc test. For Dixon's symmetry test, the overall test is performed with *𝒳*_*D*_^2^ in (18) and the post hoc tests are performed with *Z*_*D*_ in (10) for the restricted pairwise and one-versus-rest tests or *Z*_*D*_^*ij*^ for the restricted pairwise tests. For Pielou's second type of symmetry test, the overall test can be performed with *𝒳*_II_^2^ for the *k* × 3  *Q*-symmetry contingency table and post hoc tests with *𝒳*_II_^2^ for the 2 × 3  *Q*-symmetry contingency table. In the mixed or shared NN structure, significant overall tests indicate some form of deviation from symmetry for all classes combined, while the post hoc tests suggest which classes deviate significantly from symmetry. In particular, pairwise tests indicate which pairs are asymmetric in the NN structure, while one-versus-rest tests indicate which class is asymmetric with respect to the remaining classes.

In all the above cases, the post hoc tests can give different and seemingly conflicting results (e.g., one class can be symmetric with respect to the rest and at the same time it can be asymmetric with respect to one of the other classes). Even if the pairwise symmetry tests are used, the restricted or unrestricted versions might yield different results. So extra care should be exercised for which post hoc test is used and how it should be interpreted.

## 7. Example Data: Lansing Woods Data

**Figure 1:**
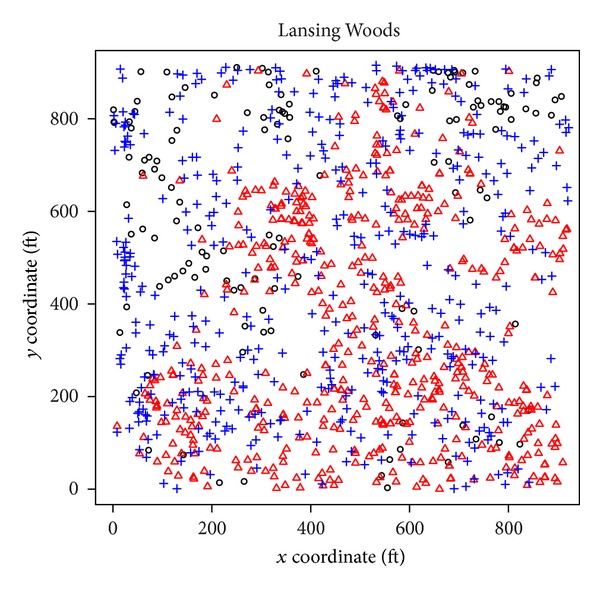
The scatter plot of the locations of black oaks (circles ∘), maples (triangles ▵), and white oaks (pluses +) in the Lansing Woods, Clinton County, MI, USA.

To illustrate the methodology, we use the Lansing Woods data, which contains locations of trees (in feet (ft)) and botanical classification of trees (according to their species) in a 924 ft × 924 ft (19.6 acre) plot in Lansing Woods, Clinton County, MI, USA [26]. The data set is available in the spatstat package in *R* [25] and comprise of 2251 trees together with their species as hickories, maples, red oaks, white oaks, black oaks, and miscellaneous trees. In our analysis, we only consider the black oaks, maples, and white oaks which constitute a total of 1097 trees. The scatter plot of these tree locations are presented in Figure 1.

### 7.1. Overall Symmetry Analysis

The NNCT for this data set is presented in Table 13. Notice that the off-diagonal entries (i.e., *N*_*ij*_ and *N*_*ji*_ values with *i* ≠ *j*) are very similar for *i* = 1, *j* = 2 and *i* = 1, *j* = 3, indicating symmetry in the mixed NN structure for black oaks versus maples and black oaks versus white oaks. But *N*_23_ and *N*_32_ values seem to be very different suggesting strong asymmetry in mixed NN structure for maples versus white oaks. We will be formally testing symmetry and attaching significance to it later in this section.

The (reduced) *Q*-symmetry contingency table is presented in Table 14, where the relative frequencies with respect to row sums are provided in parentheses. Observe that the column relative frequencies (i.e., column sums divided by the grand sum or the overall ratios of shared NNs for 0, 1, and ≥2 shared NNs) are 0.27, 0.50, and 0.24. The ratios of shared NNs for black oaks (i.e., the row entries for black oaks divided by the row sum for black oaks) are 0.27, 0.50, and 0.23, the ratios for maple trees are 0.22, 0.50, and 0.28, and the ratios for white oaks are 0.32, 0.49, and 0.19. Hence, the relative frequencies for black oaks are very similar to the overall frequencies, but those for other species (especially for white oaks) are very different from the overall frequencies. This suggests that there are differences in the shared NN structure for the three species, suggesting asymmetry in the shared NN structure, especially for white oaks compared to the other species.

We present the test statistics and the associated *p* values for the overall symmetry analysis in Table 15, where *𝒳*_*D*_^2^, *𝒳*_I_^2^, and *𝒳*_II_^2^ are as defined in the text, and the superscript *u* stands for “uncorrected for continuity” or “no Yates correction.” Furthermore, *T*_II_^*F*^ stands for the table exclusive version of two-sided Fisher's exact test on the *Q*-symmetry contingency table (which by definition only yields a *p* value but not a test statistic). Furthermore, in this table *p*_asy_ stands for the *p* value based on the asymptotic approximation (i.e., asymptotic critical value) except for the exact test; *p*_rand_ is based on Monte Carlo randomization of the labels on the given locations of the trees 1000 times. For the exact tests, the *p* value written for the *p*_asy_ row is computed as in Section 3. Notice that *p*_asy_ and *p*_rand_ are very different for Pielou's first type of symmetry test with and without Yates correction. This is in agreement with the fact that Pielou's first type of symmetry tests is not appropriate for NNCTs based on completely mapped spatial data (yielding very conservative tests under the null hypotheses). For the tests with the correct asymptotic sampling distributions, *p*_asy_ and *p*_rand_ are very similar.

Notice that the test statistics and the corresponding *p* values imply that the allocations of the tree species are asymmetric in mixed and shared NN structure (as was suggested in the NNCT and *Q*-symmetry contingency table), since the corresponding *p* values for Dixon's symmetry test and Pielou's second type of symmetry test and Fisher's exact test are significant (*p* values based on Monte Carlo randomization are significant for all tests). Hence, we will perform post hoc symmetry tests to determine which pair(s) of species or which species when compared to the rest exhibit significant asymmetry in the NN structure.

### 7.2. Post Hoc Symmetry Analysis

#### 7.2.1. Unrestricted Pairwise Symmetry Analysis

For the unrestricted pairwise analysis, we use (parts of) the contingency tables in the overall symmetry analysis. For example, for the unrestricted pairwise tests for Dixon's symmetry test, we use the off-diagonal entries *N*_*ij*_ and *N*_*ji*_ in the NNCT in Table 13 and the test statistic *Z*_*D*_^*ij*^ in (16). For the unrestricted pairwise tests for Pielou's second type of symmetry test for species *i* and *j*, we use the 2 × 3  *Q*-symmetry contingency table which is obtained by using rows *i* and *j* in Table 14.

We present the test statistics and the associated *p* values for the unrestricted pairwise symmetry tests in Table 16. Notice that *p*_asy_ and *p*_rand_ are very different for Pielou's first type of symmetry test with and without Yates correction and very similar for other tests. The test statistics and the corresponding *p* values imply that there is symmetry in NN structure for black oaks versus maples and for black oaks versus white oaks. However, maples versus white oaks exhibit significant asymmetry in NN structure.

#### 7.2.2. Restricted Pairwise Symmetry Analysis

For the restricted pairwise symmetry tests, we construct the contingency tables for the two species in question (ignoring the other species). The NNCTs for the three pairs of species are presented in Table 17. Notice that the off-diagonal entries are very similar for black oaks versus maples and black oaks versus white oaks, indicating symmetry in the mixed NN structure for these pairs of species. The off-diagonal entries are very different for maples versus white oaks indicating strong asymmetry in mixed NN structure for these species.

The (reduced) *Q*-symmetry contingency tables for each pair of species in the restricted sense are presented in Table 18, where relative frequencies of cell counts with respect to row sums are presented in parentheses. Relative frequencies of black oaks and maples seem to be very similar to the overall frequencies for the column sums, and the same holds for black oaks and white oaks, indicating symmetry in the shared NN structure. On the other hand, the relative frequencies for the maples and white oaks seem to be different from the overall frequencies, indicating asymmetry in the shared NN structure.

We present the test statistics and the associated *p* values for the restricted pairwise symmetry analysis in Table 19. Black oaks versus maples exhibit symmetry in the NN structures and likewise for black oaks versus white oaks. However, maples versus white oaks exhibit significant asymmetry in the NN structures.

#### 7.2.3. One-versus-Rest Symmetry Analysis

For the one-versus-rest type symmetry tests, we construct the contingency tables for each species in question pooling the other species in one class. The NNCTs for the three species are presented in Table 20. Notice that the off-diagonal entries are very similar for black oaks versus rest, indicating symmetry in mixed NN structure. The off-diagonal entries for maples versus rest and white oaks versus rest are very different suggesting asymmetry in mixed NN structure.

The (reduced) *Q*-symmetry contingency tables for each species in the one-versus-rest sense are presented in Table 21 which also contains the relative frequencies with respect to row sums in parentheses. The relative frequencies for black oaks versus rest are similar to the overall frequencies indicating symmetry in the shared NN structure, while they are different for maples versus rest and white oaks versus rest, indicating asymmetry in the shared NN structure.

We present the test statistics and the associated *p* values for the one-versus-rest symmetry analysis in Table 22. There is symmetry in NN structures for black oaks versus rest, while significant asymmetry in NN structures for maples versus rest and white oaks versus rest.

## 8. Interpoint Dissimilarity Measures

A *dissimilarity measure*, *ρ*, on a set of objects *E* is the ℝ valued function on *E* × *E* such that *ρ*_x_* = *ρ*(*x*, *x*) ≤ *ρ*(*x*, *y*) = *ρ*(*y*, *x*) < *∞* for all *x*, *y* ∈ *E*. A *similarity measure*, *s*, on *E* is the ℝ valued function on *E* × *E* such that *s*_x_* = *s*(*x*, *x*) ≥ *s*(*x*, *y*) = *s*(*y*, *x*) ≥ 0 for all *x*, *y* ∈ *E*. Generally, *ρ*_x_* = ρ* and *s*_x_* = s* for all *x* ∈ *E*. In particular, if s* = 0, then ρ* = 1. We focus on dissimilarity measures only, since any similarity measure can easily be converted to a dissimilarity measure [27]. Any distance metric is by definition a dissimilarity measure. In practice, the term distance is often used to describe precisely the differences of actual measurements, while “dissimilarity” might be an estimation of a distance we can not measure physically. Among the widely used distances are Euclidean, Minkowski, Mahalanobis, and taxi-cab distances; among the nonmetric dissimilarity measures are maximum coordinate difference, minimum coordinate difference, dot product, Pearson's linear dissimilarity, and Spearman's rank dissimilarity.

In the literature usually NN relationships defined with distance metrics are used. In particular, Euclidean distance in ℝ^2^ is the only metric used in this paper. The use of distances for obtaining the NN relations can be generalized to dissimilarity measures in such a way that the NN of an object, *x*, refers to the object with the minimum dissimilarity to *x*. We assume that the objects (events) lie in a finite or infinite dimensional space satisfying the symmetry conditions. Under RL, the objects are fixed in the sense that they yield fixed interpoint dissimilarity measures, but the labels are assigned randomly.

The spatial patterns have broader interpretations in this extension. Symmetry occurs when the classes have similar NN structures with respect to each other. The extension of Pielou's first type of symmetry test is straightforward. However, Pielou's second type of symmetry test and Dixon's tests are constructed assuming that data are in ℝ^2^ in the literature. In Dixon's tests, the term *Q* which is the number of points with shared NNs needs to be updated for higher dimensional data. The general form of *Q* is defined as $\tilde{Q}:=2\sum_{j=1}^{n}\left(\begin{array}{c} j\\ 2 \end{array}\right)‍Q_{j}$. In practice, usually $\tilde{Q}\approx Q$. One may check the appropriateness of this assumption by using the interpoint dissimilarity matrix in the classical multidimensional scaling of the data to ℝ^2^. If the NN relations do not change considerably, it might be more practical to just use *Q* instead of $\tilde{Q}$ for computational reasons. Furthermore, with non-Euclidean distances or dissimilarity measures, a point can serve as a NN to more than 6 points, so the *Q*-symmetry contingency table should be updated accordingly.

Here is a possible example for which we have dissimilarity measures between objects that lie in a high or infinite dimensional space. In medical image analysis the differences in morphometry (shape and size) of tissues are measured by a distance metric called LDDMM (see, e.g., [28]). Based on the distances measured between certain brain tissues (like hippocampus), one is interested, say, in the symmetry of the shapes of the tissues with respect to NN relationships. This aspect of spatial dependence in the (abstract) morphometric space is a topic of prospective research.

## 9. Discussion and Conclusions

In this paper, we investigate tests of symmetry in mixed and shared nearest neighbor (NN) structures using contingency tables based on the NN relations between classes. We consider Pielou's two types of symmetry tests and Dixon's symmetry test and determine their appropriate null hypotheses and the underlying assumptions. Pielou's first type of symmetry test and Dixon's symmetry tests are for symmetry in mixed NN structure and are based on the nearest neighbor contingency table (NNCT), while Pielou's second type of symmetry test is for symmetry in shared NN structure and is based on the *Q*-symmetry contingency table. We derive the asymptotic distribution of Dixon's symmetry test under RL, which is also valid under CSR independence conditional on spatial allocation of the points in the study region. We extend Pielou's and Dixon's symmetry tests to multiclass case and prove the consistency of these tests under their appropriate null hypotheses. In particular, we prove consistency for Pielou's first type of symmetry test under the appropriate sparse sampling in the overall multinomial framework, for Pielou's second type of symmetry test under the appropriate sparse sampling in the row-wise multinomial framework and for Dixon's symmetry test under RL patterns with completely mapped data.

Among the symmetry tests, we demonstrate that versions of Pielou's first type of symmetry test are extremely conservative when used with the asymptotic critical value for the McNemar's test, due to dependence between base-NN pairs and the underlying framework for the NNCT. Hence, these tests should be avoided in practice with the asymptotic critical values but can be used with Monte Carlo randomization. On the other hand, Pielou's second type of symmetry test and Dixon's symmetry test are about the desired level under complete spatial randomness (CSR) independence and random labeling (RL). We also consider the use of Fisher's exact test for the *Q*-symmetry contingency table. In particular, we demonstrate that the table exclusive version of the two-sided exact test has the desired level under CSR independence and RL. It is also desirable for a test not only to be consistent but also powerful; hence, determining appropriate alternatives for these tests is an important task. We consider various patterns for assessing the finite sample performance of symmetry tests and discover other patterns under which the null hypotheses for these types of symmetry tests are satisfied. However, the variances and covariances (and hence the asymptotic distributions) should be adjusted to have the desired level for these patterns, because the asymptotic distribution of Dixon's symmetry test is only derived under CSR independence and RL. With the critical values based on the asymptotic distribution under CSR independence or RL, the tests are either extremely conservative or liberal (although the null hypotheses are satisfied). We also find that some of the patterns can serve as alternatives for symmetry in shared NN structure or for symmetry in mixed NN structure. Under these alternatives, we observe that Pielou's second type of symmetry test has higher power compared to Dixon's test of symmetry. Furthermore, Pielou's second type of symmetry test is only empirically shown to be appropriate under CSR independence and RL by Monte Carlo simulations. Finding the distribution of Dixon's symmetry test under the null hypothesis of symmetry in mixed NN structure in general (as CSR independence and RL are only two special cases in this setting) and finding the distribution of Pielou's second type of symmetry under the null hypothesis of symmetry in shared NN structure (even under CSR independence or RL) are still open problems.

In a multiclass setting, first an overall symmetry test can be conducted as an omnibus test. If significant, then either one-versus-rest or pairwise type post hoc tests can be applied. If the interest is in the symmetry of one class with respect to the remaining classes, then a one-versus-rest type analysis should be performed. On the other hand, if the interest is in determining which pair(s) significantly deviate from symmetry, then pairwise symmetry tests can be employed. When we are doing the pairwise tests after an overall symmetry test, we recommend the unrestricted pairwise version, which takes all the data into account (indeed the significant overall test was based on all the data considered). But if the interest is only on two of the classes, then a restricted pairwise test (considering only the classes in question) can be employed. For symmetry in shared NN structure (with *Q*-symmetry contingency table), we recommend the use of one-versus-rest type post hoc tests, as they are more consistent with the overall symmetry test.

Throughout the paper, we assumed that the total sample size *n* is a fixed quantity. To make it a random variable, one may consider that data are from a Poisson point process over the (bounded) region of interest. The generalizations of the tests to high dimensional data and NNCTs based on general dissimilarity measures make this methodology useful for other fields as well.

Finally, the tests in this paper are not adjusted for the influence of the edges or boundary of the support, which usually causes the tests to be slightly liberal or conservative. Such an adjustment is only necessary when the spatial allocation of the points is not fixed but results from a stochastic process whose support contains the study region (as in the CSR independence case). To make the size of the test appropriate, the tests need to be adjusted for boundary effects, which is also a topic of prospective research.

## Figures and Tables

**Table 1:** The NNCT for two classes.

	NN class		Total
	Class 1	Class 2	
Base class			
Class 1	*N*_11_	*N*_12_	*n*_1_
Class 2	*N*_21_	*N*_22_	*n*_2_

Total	*C*_1_	*C*_2_	*n*

**Table:** (a)

	The number of times a point serving as a NN						Total
	0	1	2	3	4	5	
Classes							
Class 1	*Q*_1,0_	*Q*_1,1_	*Q*_1,2_	*Q*_1,3_	*Q*_1,4_	*Q*_1,5_	*n*_1_
Class 2	*Q*_2,0_	*Q*_2,1_	*Q*_2,2_	*Q*_2,3_	*Q*_2,4_	*Q*_2,5_	*n*_2_

Total	*Q*_0_	*Q*_1_	*Q*_2_	*Q*_3_	*Q*_4_	*Q*_5_	*n*

**Table:** (b)

	The number of times a point serving as a NN						Total
	0	1	2	3	4	5	
Classes							
Class 1	*Q*_1,0_	*Q*_1,1_	*Q*_1,2_	*Q*_1,3_	*Q*_1,4_	*Q*_1,5_	*n*_1_
⋮	⋮	⋮	⋮	⋮	⋮	⋮	⋮
Class *k*	*Q*_*k*,0_	*Q*_*k*,1_	*Q*_*k*,2_	*Q*_*k*,3_	*Q*_*k*,4_	*Q*_*k*,5_	*n*_*k*_

Total	*Q*_0_	*Q*_1_	*Q*_2_	*Q*_3_	*Q*_4_	*Q*_5_	*n*

**Table 3:** The empirical significance levels of the symmetry tests under CSR independence Case  1: *n*_1_ = *n*_2_ = *n* = 10,20,…, 50 and Case  2: *n*_1_ = 20, *n*_2_ = 20,30,…, 60 with *N*_mc_ = 10000 at *α* = .05. ${\hat{\alpha }}_{\text{I}}^{P}$ and ${\hat{\alpha }}_{\text{I}}^{P^{\prime }}$ stand for the empirical significance levels for Pielou's first type of symmetry test using *χ* ^2^ approximation with and without Yates' continuity correction, respectively; ${\hat{\alpha }}_{\text{bin}}^{P}$ stands for the exact binomial version of Pielou's first type of symmetry test conditional on *N*_12_ + *N*_21_ = *n*_*t*_; ${\hat{\alpha }}_{\text{II}}^{P}$ stands for the empirical significance level for Pielou's second type of symmetry test; ${\hat{\alpha }}_{S}^{D}$ stands for Dixon's symmetry test.

CSR independence Case 1					
*n*	${\hat{\alpha }}_{\text{I}}^{P}$	${\hat{\alpha }}_{\text{I}}^{P^{\prime }}$	${\hat{\alpha }}_{\text{bin}}^{P}$	${\hat{\alpha }}_{\text{II}}^{P}$	${\hat{\alpha }}_{S}^{D}$
10	.0002	.0011	.0111	.0483	.0466
20	.0001	.0006	.0080	.0533	.0480
30	.0000	.0008	.0073	.0487	.0492
40	.0001	.0006	.0061	.0501	.0514
50	.0000	.0007	.0044	.0522	.0484

CSR independence Case 2					
*n*_2_	${\hat{\alpha }}_{\text{I}}^{P}$	${\hat{\alpha }}_{\text{I}}^{P^{\prime }}$	${\hat{\alpha }}_{\text{bin}}^{P}$	${\hat{\alpha }}_{\text{II}}^{P}$	${\hat{\alpha }}_{S}^{D}$

20	.0001	.0006	.0093	.0533	.0473
30	.0002	.0001	.0088	.0527	.0500
40	.0001	.0008	.0089	.0536	.0539
50	.0002	.0008	.0092	.0491	.0483
60	.0002	.0010	.0080	.0508	.0483

**Table 4:** The empirical significance levels for Fisher's two-sided exact tests for the *Q*-symmetry contingency tables under CSR independence Cases 1 and 2 with *N*_mc_ = 10000, for some combinations of *n*_1_, *n*_2_ at *α* = .05. ${\hat{\alpha }}_{\text{inc}}$ is for the empirical significance level for the table-inclusive version of the two-sided test, ${\hat{\alpha }}_{\text{exc}}$ is for table-exclusive version, ${\hat{\alpha }}_{\text{mid}}$ is for mid-*p* value version, and ${\hat{\alpha }}_{\text{Toc}}$ is for Tocher corrected version.

CSR independence Case 1					
n	${\hat{\alpha }}_{\text{inc}}$	${\hat{\alpha }}_{\text{exc}}$	${\hat{\alpha }}_{\text{mid}}$	${\hat{\alpha }}_{\text{Toc}}$	${\hat{\alpha }}_{t,\text{inc}}$
10	.0392	.0424	.0413	.0410	.0319
20	.0466	.0505	.0480	.0487	.0451
30	.0459	.0484	.0465	.0466	.0430
40	.0457	.0481	.0475	.0472	.0434
50	.0475	.0490	.0478	.0479	.0462

CSR independence Case 2					
*n*_2_	${\hat{\alpha }}_{\text{inc}}$	${\hat{\alpha }}_{\text{exc}}$	${\hat{\alpha }}_{\text{mid}}$	${\hat{\alpha }}_{\text{Toc}}$	${\hat{\alpha }}_{t,\text{inc}}$

20	.0454	.0497	.0462	.0469	.0444
30	.0484	.0533	.0504	.0512	.0435
40	.0479	.0505	.0489	.0490	.0460
50	.0485	.0524	.0504	.0509	.0460
60	.0474	.0500	.0489	.0487	.0445

**Table 5:** The empirical significance levels of the tests under RL Cases  1–3 with *N*_mc_ = 1000 for each of 100 background realization at *α* = .05. The empirical size labeling is as in Table 3. ${\hat{\alpha }}_{\text{II}}^{F}$ stands for the empirical size estimates of the exact tests for Pielou's second type of symmetry (the table exclusive version).

RL Case 1						
*n*	${\hat{\alpha }}_{\text{I}}^{P}$	${\hat{\alpha }}_{\text{I}}^{P^{\prime }}$	${\hat{\alpha }}_{\text{bin}}^{P}$	${\hat{\alpha }}_{S}^{D}$	${\hat{\alpha }}_{\text{II}}^{P}$	${\hat{\alpha }}_{\text{II}}^{F}$
10	.00011	.00089	.00092	.04368	.04989	.04205
20	.00018	.00096	.00106	.04797	.05283	.04831
30	.00017	.00095	.00110	.05037	.05147	.04974
40	.00016	.00056	.00042	.05156	.05242	.04994
50	.00028	.00070	.00050	.04981	.05020	.04934

RL Case 2						
*n*_2_	${\hat{\alpha }}_{\text{I}}^{P}$	${\hat{\alpha }}_{\text{I}}^{P^{\prime }}$	${\hat{\alpha }}_{\text{bin}}^{P}$	${\hat{\alpha }}_{S}^{D}$	${\hat{\alpha }}_{\text{II}}^{P}$	${\hat{\alpha }}_{\text{II}}^{F}$

20	.00016	.00089	.00109	.04907	.05298	.04987
30	.00016	.00090	.00063	.05087	.05143	.05258
40	.00015	.00069	.00048	.04880	.05087	.05222
50	.00026	.00099	.00079	.04700	.05010	.05271
60	.00027	.00097	.00084	.04991	.04985	.05104

RL Case 3						
*κ*	${\hat{\alpha }}_{\text{I}}^{P}$	${\hat{\alpha }}_{\text{I}}^{P^{\prime }}$	${\hat{\alpha }}_{\text{bin}}^{P}$	${\hat{\alpha }}_{S}^{D}$	${\hat{\alpha }}_{\text{II}}^{P}$	${\hat{\alpha }}_{\text{II}}^{F}$

2	.00018	.00063	.00065	.05158	.05241	.05006
4	.00023	.08451	.00083	.04848	.05024	.04988
6	.00012	.00051	.00048	.04953	.05061	.05057
8	.00024	.00076	.00074	.05075	.05007	.04963
10	.00024	.00070	.00083	.04939	.05087	.05063

**Table:** (a)

	Mean ± SD			Rejection rate estimates for Case I patterns					
	*N*_12_	*N*_21_	*N*_12_ − *N*_21_	${\hat{\beta }}_{\text{I}}^{P}$	${\hat{\beta }}_{\text{I}}^{P^{\prime }}$	${\hat{\beta }}_{\text{bin}}^{P}$	${\hat{\beta }}_{S}^{D}$	${\hat{\beta }}_{\text{II}}^{P}$	${\hat{\beta }}_{\text{II}}^{F}$
I-(i)	8.5 ± 2.5	6.8 ± 2.7	1.66 ± 2.26	.0018	.0060	.0721	.0092	.0379	.0359
I-(ii)	4.1 ± 1.7	2.2 ± 1.7	1.94 ± 1.58	.0118	.0511	.3109	.0017	.0345	.0324
I-(iii)	2.9 ± 1.4	1.0 ± 1.2	1.87 ± 1.34	.0338	.0919	.5345	.0002	.0324	.0296

**Table:** (b)

	Rejection rate estimates for Case I patterns based on Monte Carlo randomization				
	${\hat{\beta }}_{\text{I}}^{P}$	${\hat{\beta }}_{\text{I}}^{P^{\prime }}$	${\hat{\beta }}_{S}^{D}$	${\hat{\beta }}_{\text{II}}^{P}$	${\hat{\beta }}_{\text{II}}^{F}$
I-(i)	.641219	.480581	.409682	.546873	.546491
I-(ii)	.670268	.402391	.271784	.569909	.569605
I-(iii)	.696817	.397304	.196840	.577398	.576899

**Table 7:** The means (±SD) of the off-diagonal entries, *N*_12_, *N*_21_, and their difference *N*_12_ − *N*_21_ under CSR independence Case  1 and RL Case  1 with *n*_1_ = *n*_2_ = 40 at *α* = .05.

	Mean ± SD		
	*N*_12_	*N*_21_	*N*_12_ − *N*_21_
CSR-ind Case 1	20.2 ± 3.3	20.3 ± 3.4	− .03 ± 3.60
RL Case 1	20.3 ± 3.4	20.3 ± 3.4	− .01 ± 3.57

**Table 8:** The means (±SD) of the off-diagonal entries, *N*_12_, *N*_21_, and their difference *N*_12_ − *N*_21_ and the rejection rate estimates for Case II patterns in (34) with *N*_mc_ = 10000, *n*_1_ = *n*_2_ = 40 at *α* = .05. Column labeling is as in Table 6.

	Mean ± SD			Rejection rate estimates for Case II patterns					
	*N*_12_	*N*_21_	*N*_12_ − *N*_21_	${\hat{\beta }}_{\text{I}}^{P}$	${\hat{\beta }}_{\text{I}}^{P^{\prime }}$	${\hat{\beta }}_{\text{bin}}^{P}$	${\hat{\beta }}_{S}^{D}$	${\hat{\beta }}_{\text{II}}^{P}$	${\hat{\beta }}_{\text{II}}^{F}$
II-(i)	28.0 ± 3.9	27.3 ± 3.5	.68 ± 3.80	.0002	.0002	.0030	.0198	.3313	.3225
II-(ii)	36.4 ± 4.4	33.5 ± 3.0	2.84 ± 4.07	.0001	.0009	.0084	.0124	.8395	.8326
II-(iii)	45.3 ± 4.6	38.1 ± 1.8	7.21 ± 4.43	.0049	.0079	.0524	.0434	.9923	.9913

**Table 9:** The means (±SD) of the off-diagonal entries, *N*_12_, *N*_21_, and their difference *N*_12_ − *N*_21_ and the rejection rate estimates for Case III patterns with *N*_mc_ = 10000, *n*_1_ = *n*_2_ = 40 at *α* = .05. Column labeling is as in Table 6.

	Mean ± SD			Rejection rate estimates for Case III patterns					
	*N*_12_	*N*_21_	*N*_12_ − *N*_21_	${\hat{\beta }}_{\text{I}}^{P}$	${\hat{\beta }}_{\text{I}}^{P^{\prime }}$	${\hat{\beta }}_{\text{bin}}^{P}$	${\hat{\beta }}_{S}^{D}$	${\hat{\beta }}_{\text{II}}^{P}$	${\hat{\beta }}_{\text{II}}^{F}$
III-(i)	14.4 ± 3.1	14.4 ± 3.1	.04 ± 3.09	.0004	.0009	.0086	.0225	.0385	.0368
III-(ii)	10.1 ± 2.7	10.1 ± 2.7	− .01 ± 2.58	.0000	.0011	.0096	.0073	.0341	.0321
III-(iii)	5.9 ± 2.1	5.9 ± 2.1	.03 ± 1.98	.0002	.0013	.0128	.0005	.0336	.0308

**Table 10:** The means (±SD) of the off-diagonal entries, *N*_12_, *N*_21_, and their difference *N*_12_ − *N*_21_ and the rejection rate estimates for Case IV patterns with *N*_mc_ = 10000, *n*_1_ = *n*_2_ = 40 at *α* = .05. The rejection rate labeling and superscripting for “<” and “>” are as in Table 6.

	*r*	Mean ± SD			Rejection rate estimates for Case IV patterns					
		*N*_12_	*N*_21_	*N*_12_ − *N*_21_	${\hat{\beta }}_{\text{I}}^{P}$	${\hat{\beta }}_{\text{I}}^{P^{\prime }}$	${\hat{\beta }}_{\text{bin}}^{P}$	${\hat{\beta }}_{S}^{D}$	${\hat{\beta }}_{\text{II}}^{P}$	${\hat{\beta }}_{\text{II}}^{F}$
IV-(i)	1/7	10.5 ± 3.2	10.5 ± 3.2	− .05 ± 3.12	.0012	.0055	.0256	.0361	.0565	.0525
	1/8	9.3 ± 3.1	9.3 ± 3.1	− .03 ± 2.99	.0021	.0052	.0307	.0318	.0572	.0552
	1/9	8.2 ± 3.0	8.2 ± 3.0	.00 ± 2.86	.0023	.0071	.0351	.0295	.0579	.0562

IV-(ii)	1/7	9.0 ± 3.0	9.0 ± 3.1	− .01 ± 2.78	.0014	.0039	.0235	.0176	.0526	.0509
	1/8	8.1 ± 3.0	8.1 ± 3.0	− .02 ± 2.70	.0015	.0064	.0312	.0192	.0609	.0583
	1/9	7.2 ± 2.9	7.2 ± 2.9	− .01 ± 2.63	.0028	.0075	.0395	.0172	.0616	.0601

IV-(iii)	1/7	6.9 ± 2.9	6.9 ± 2.8	.01 ± 2.42	.0014	.0039	.0286	.0070	.0496	.0470
	1/8	6.3 ± 2.8	6.3 ± 2.7	.02 ± 2.37	.0020	.0061	.0345	.0094	.0539	.0518
	1/9	5.6 ± 2.7	5.6 ± 2.6	.01 ± 2.30	.0027	.0074	.0392	.0070	.0590	.0565

**Table 11:** The means (±SD) of the off-diagonal entries, *N*_12_, *N*_21_, and their difference *N*_12_ − *N*_21_ and the rejection rate estimates for Case V patterns with *N*_mc_ = 10000, *n*_1_ = *n*_2_ = 40 at *α* = .05. Column labeling is as in Table 6.

	Mean ± SD			Rejection rate estimates for Case V patterns					
	*N*_12_	*N*_21_	*N*_12_ − *N*_21_	${\hat{\beta }}_{\text{I}}^{P}$	${\hat{\beta }}_{\text{I}}^{P^{\prime }}$	${\hat{\beta }}_{\text{bin}}^{P}$	${\hat{\beta }}_{S}^{D}$	${\hat{\beta }}_{\text{II}}^{P}$	${\hat{\beta }}_{\text{II}}^{F}$
V-(i)	19.5 ± 3.3	21.9 ± 3.2	− 2.44 ± 3.52	.0004	.0019	.0076	.0982	.0805	.0982
V-(ii)	22.9 ± 3.3	24.5 ± 3.2	− 1.61 ± 3.62	.0002	.0007	.0033	.0752	.0699	.0676
V-(iii)	25.9 ± 3.1	26.5 ± 3.1	− .60 ± 3.58	.0001	.0003	.0021	.0565	.0503	.0490
V-(iv)	27.8 ± 2.9	27.9 ± 3.0	− .01 ± 3.52	.0001	.0002	.0012	.0513	.0485	.0454

**Table 12:** The means (±SD) of the off-diagonal entries, *N*_12_, *N*_21_, and their difference *N*_12_ − *N*_21_ and the rejection rate estimates for Case VI patterns with *N*_mc_ = 10000, *m*_1_ = 20, *m*_2_ = 10 (hence *n*_1_ = *n*_2_ = 40) at *α* = .05. Column labeling is as in Table 6.

	Mean ± SD			Rejection rate estimates for Case VI patterns					
	*N*_12_	*N*_21_	*N*_12_ − *N*_21_	${\hat{\beta }}_{\text{I}}^{P}$	${\hat{\beta }}_{\text{I}}^{P^{\prime }}$	${\hat{\beta }}_{\text{bin}}^{P}$	${\hat{\beta }}_{S}^{D}$	${\hat{\beta }}_{\text{II}}^{P}$	${\hat{\beta }}_{\text{II}}^{F}$
VI-(i)	20.3 ± .6	29.0 ± 2.6	− 8.73 ± 2.58	.0019	.0091	.0629	.8883	.9911	.9907
VI-(ii)	21.6 ± 1.4	27.7 ± 2.8	− 6.13 ± 2.89	.0003	.0012	.0073	.5460	.7782	.7730
VI-(iii)	23.1 ± 2.1	26.4 ± 3.0	− 3.39 ± 3.25	.0000	.0000	.0009	.1860	.3029	.2980

**Table 13:** The NNCT for the Lansing Woods data set containing black oak, maple, and white oak trees.

	NN species			Total
	Black oak	Maple	White oak	
Base species				
Black oak	53	35	47	135
Maple	28	366	120	514
White oak	50	161	237	448

Total	131	562	404	1097

**Table 14:** The (reduced) *Q*-symmetry contingency table for the Lansing Woods data. The values in the parentheses are relative frequencies of the cells in each row with respect to the row sums.

	Number of times a point serving as a NN			Total
	0	1	≥2	
Classes				
Black oak	37 (.27)	67 (.50)	31 (.23)	135
Maple	113 (.22)	259 (.50)	142 (.28)	514
White oak	143 (.32)	220 (.49)	85 (.19)	448

Total	293 (.27)	546 (.50)	258 (.24)	1097

**Table 15:** The test statistics and the *p*-values for the overall symmetry analysis for the Lansing Woods data. TS stands for the test statistic, *p*_asy_ for the *p*-values based on asymptotic critical values (except for the exact tests), and *p*_rand_ for the *p*-values based on Monte Carlo randomization. *The *p*-values for the exact tests are not the asymptotic *p*-values but computed as described in Section 3.

Overall test statistics and *p*-values					
	*𝒳*_*D*_^2^	*𝒳*_I_^2^	*𝒳*_I_^2,*u*^	*𝒳*_II_^2^	*T*_II_^*F*^
TS	13.482	5.694	6.182	16.595	—
*p*_asy_	.004	.128	.103	.002	.002*
*p*_rand_	.006	.002	.002	.004	.004

**Table 16:** The test statistics and the *p*-values for the unrestricted pairwise symmetry analysis for the Lansing Woods data. Row labelings and the asterisks are in Table 15.

Unrestricted pairwise test statistics and *p*-values					
	*Z*_*D*_^*ij*^	*𝒳*_I_^2^	*𝒳*_I_^2,*u*^	*𝒳*_II_^2^	*T*_II_^*F*^
Black oaks versus maples					
TS	.769	.246	.385	2.245	—
*p*_asy_	.442	.620	.535	.326	.331*
*p*_rand_	.412	.311	.282	.331	.337

Black oaks versus white oaks					
TS	−.657	.092	.163	1.520	—
*p*_asy_	.511	.762	.686	.468	.466*
*p*_rand_	.473	.483	.483	.481	.481

Maples versus white oaks					
TS	−3.470	5.356	5.634	16.554	—
*p*_asy_	<.001	.021	.018	<.001	<.001*
*p*_rand_	<.001	<.001	<.001	<.001	<.001

**Table:** (a)

	NN species		Total
	B.O.	Maple	
Base species			
B.O.	82	53	135
Maple	49	465	514

Total	131	518	649

**Table:** (b)

	NN species		Total
	B.O.	W.O.	
B.O.	78	67	135
W.O.	72	376	448

Total	140	443	583

**Table:** (c)

	NN species		Total
	Maple	W.O.	
Maple	379	135	514
W.O.	172	276	448

Total	551	411	962

**Table:** (a)

	Number of times a point serving as a NN			Total
	0	1	≥2	
Classes				
B.O.	38 (.28)	65 (.48)	32 (.24)	135
M.	142 (.28)	242 (.47)	130 (.25)	514

Total	180 (.28)	307 (.47)	162 (.25)	649

**Table:** (b)

	Number of times a point serving as a NN			Total
	0	1	≥2	
B.O.	36 (.27)	64 (.47)	35 (.26)	135
W.O.	135 (.30)	203 (.45)	110 (.25)	448

Total	171 (.29)	267 (.46)	145 (.25)	583

**Table:** (c)

	Number of times a point serving as a NN			Total
	0	1	≥2	
M.	117 (.23)	258 (.50)	139 (.27)	514
W.O.	136 (.30)	224 (.50)	88 (.20)	448

Total	253 (.26)	482 (.50)	227 (.24)	962

**Table 19:** The test statistics and the *p*-values for the unrestricted pairwise symmetry analysis for the Lansing Woods data. Row labelings and the asterisks are in Table 15.

Restricted pairwise test statistics and *p*-values					
	*Z*_*D*_^*ij*^	*𝒳*_I_^2^	*𝒳*_I_^2,*u*^	*𝒳*_II_^2^	*T*_II_^*F*^
Black oaks versus maples					
TS	.246	.010	.038	.144	—
*p*_asy_	.806	.922	.845	.930	.937*
*p*_rand_	.819	.778	.773	.932	.945

Black oaks versus white oaks					
TS	−.597	.115	.180	.603	—
*p*_asy_	.551	.734	.671	.740	.759*
*p*_rand_	.598	.497	.455	.764	.778

Maples versus white oaks					
TS	−2.923	3.717	3.939	10.806	—
*p*_asy_	.003	.054	.047	.005	.005*
*p*_rand_	<.001	<.001	<.001	.004	.004

**Table:** (a)

	NN species		Total
	B.O.	Rest	
Base species			
B.O.	53	82	135
Rest	78	884	964

Total	131	966	1097

**Table:** (b)

	NN species		Total
	Maple	Rest	
Maple	352	150	514
Rest	196	387	583

Total	560	537	1097

**Table:** (c)

	NN species		Total
	W.O.	Rest	
W.O.	236	212	448
Rest	167	482	649

Total	403	694	1097

**Table:** (a)

	Number of times a point serving as a NN			Total
	0	1	≥2	
Classes				
B.O.	37 (.27)	67 (.50)	31 (.23)	135
R.	256 (.27)	479 (.50)	227 (.24)	962

Total	293 (.27)	546 (.50)	258 (.24)	1097

**Table:** (b)

	Number of times a point serving as a NN			Total
	0	1	≥2	
M.	112 (.22)	259 (.50)	148 (.29)	514
R.	181 (.31)	286 (.49)	116 (.20)	583

Total	293 (.27)	545 (.50)	259 (.24)	1097

**Table:** (c)

	Number of times a point serving as a NN			Total
	0	1	≥2	
W.O.	143 (.32)	219 (.49)	86 (.19)	448
R.	150 (.23)	327 (.50)	172 (.27)	649

Total	293 (.27)	546 (.50)	258 (.24)	1097

**Table 22:** The test statistics and the *p*-values for the unrestricted pairwise symmetry analysis for the Lansing Woods data. Row labelings and the asterisks are in Table 15.

One-versus-rest test statistics and *P*-values					
	*Z*_*D*_^*ij*^	*𝒳*_I_^2^	*𝒳*_I_^2,*u*^	*𝒳*_II_^2^	*T*_II_^*F*^
Black oak versus rest					
TS	.118	.000	.006	.049	—
*p*_asy_	.906	1.000	.938	.976	.970*
*p*_rand_	.889	1.000	.866	.968	.968

Maples versus rest					
TS	−3.471	5.547	5.802	16.125	—
*p*_asy_	<.001	.019	.016	<.001	<.001*
*p*_rand_	<.001	<.001	<.001	<.001	<.001

White oaks versus rest					
TS	3.447	4.840	5.068	13.832	—
*p*_asy_	<.001	.028	.024	<.001	<.001*
*p*_rand_	.001	<.001	<.001	.002	.002

## Appendix

### Proof of Theorem 7

Let *C* ^2^ ~ *χ*_*ν*_^2^(*λ*). Then the pdf of *C* ^2^ is

(A.1) $$\begin{array}{c} f_{\nu }\left(x,\lambda \right)=\frac{\exp\left(-\left(\nu +\lambda \right)/2\right)x^{\nu /2-1}}{2^{\nu /2}}\overset{\infty }{\underset{j=0}{\sum }}\frac{{\left(x\lambda /4\right)}^{j}}{\Gamma \left(\nu /2+j\right)j!} \end{array}$$

for *λ* > 0, *ν* a positive integer, and *x* > 0 with the conventions of 0! = 0^0^ = 1. Then **E**[*C* ^2^] = *ν* + *λ* and **V** **a** **r**[*C* ^2^] = 2(*ν* + 2  *λ*). The power at level *α* is

(A.2) β(λ,ν,α)=P(C2>χν2(λ=0,1−α))=∫χν2(0,1−α)∞fν(x,λ)dx.

The Taylor series expansion of *β*(*λ*, *ν*, *α*) around *λ* = 0 for fixed *α* and *ν* is given by

(A.3) β(λ,ν,α)=∑k=0∞λkβ(k)(λ=0,ν,α)k!=β(0,ν,α)+λβ′(0,ν,α)+R(λ,ν,α),

where *R*(*λ*, *ν*, *α*) = *O*(*λ* ^2^) as *λ* → 0. Note that

(A.4) $$\begin{array}{c} \beta \left(0,\nu ,\alpha \right)=\overset{\infty }{\underset{\chi_{\nu }^{2}\left(0,1-\alpha \right)}{\int }}f_{\nu }\left(x,\lambda \right)dx=\alpha , \end{array}$$

since under *H*_*o*_ power equals the level of the test. Next,

(A.5) $$\begin{array}{c} \beta^{\prime }\left(0,\nu ,\alpha \right)=\overset{\infty }{\underset{\chi_{\nu }^{2}(0,1-\alpha )}{\int }}\frac{\partial f_{\nu }\left(x,\lambda \right)}{\partial \lambda }dx \end{array}$$

(see, e.g., [29]), where

(A.6) ∂fν(x,λ)∂λ=−12fν(x,λ)+exp⁡(−(x+λ)/2)xν/2−12ν/2×∑k=0∞k(x/4)kλk−1Γ(ν/2+k)k!.

Substituting *λ* = 0, we get

(A.7) ∂fν(x,λ)∂λ|λ=0=−12fν(x,0)+exp⁡(−x/2)xν/2−12ν/2·x4=−12fν(x,0)+12fν+2(x,0).

So *β*′(0, *ν*, *α*) = −(*α*/2)+(1/2)[1 − *F*_*ν*+2_(*χ*_*ν*_^2^(0,1 − *α*))]. Thus, for small *λ* > 0,

(A.8) $$\begin{array}{c} \beta \left(\lambda ,\nu ,\alpha \right)\approx \alpha +\lambda \left(-\frac{\alpha }{2}+\frac{1}{2}\left[1-F_{\nu +2}\left(\chi_{\nu }^{2}\left(0,1-\alpha \right)\right)\right]\right).\; \end{array}$$
